# Expression of Dopamine-Related Genes in Four Human Brain Regions

**DOI:** 10.3390/brainsci10080567

**Published:** 2020-08-18

**Authors:** Ansley Grimes Stanfill, Xueyuan Cao

**Affiliations:** 1 Associate Professor and Associate Dean of Research, College of Nursing, University of Tennessee Health Science Center, Memphis, TN 38163, USA; 2 Assistant Professor, College of Nursing, University of Tennessee Health Science Center, Memphis, TN 38163, USA; xcao12@uthsc.edu

**Keywords:** dopamine, dopamine receptors, mesocortical, mesolimbic, nigrostrial, prefrontal cortex, nucleus accumbens, substantia nigra, hippocampus, human

## Abstract

A better understanding of dopaminergic gene expression will inform future treatment options for many different neurologic and psychiatric conditions. Here, we utilized the National Institutes of Health’s Genotype-Tissue Expression project (GTEx) dataset to investigate genotype by expression associations in seven dopamine pathway genes (*ANKK1*, *DBH*, *DRD1*, *DRD2*, *DRD3*, *DRD5*, and *SLC6A3*) in and across four human brain tissues (prefrontal cortex, nucleus accumbens, substantia nigra, and hippocampus). We found that age alters expression of *DRD1* in the nucleus accumbens and prefrontal cortex, *DRD3* in the nucleus accumbens, and *DRD5* in the hippocampus and prefrontal cortex. Sex was associated with expression of *DRD5* in substantia nigra and hippocampus, and *SLC6A3* in substantia nigra. We found that three linkage disequilibrium blocks of SNPs, all located in *DRD2*, were associated with alterations in expression across all four tissues. These demographic characteristic associations and these variants should be further investigated for use in screening, diagnosis, and future treatment of neurological and psychiatric conditions.

## 1. Introduction

Multiple neurological and psychiatric diseases result from alterations in the production, degradation, or improper signaling of dopamine in the brain. In order to develop better therapeutic targets for these diseases, we must first better understand the organization and connectivity of dopaminergic circuitry in the brain. Within the mesocortical and mesolimbic pathways, the intricate balance of dopaminergic gene expression controls much of the reward and motivation machinery in the brain. Such a balance in the nigrostrial pathway influences movement and procedural learning, while the hippocampus is involved in long-term memory, spatial processing, and navigation. An imbalance of dopamine expression in any of these areas can create any number of heterogenous behavioral outcomes, including many common neurological and psychiatric conditions. Such an imbalance is often due to genotypic variations that alter expression. Although much work has been done in this area using animal models, it is not yet clearly understood in humans how dopaminergic expression (a) changes across the regions of such circuitry and (b) varies by given genotype. 

One way to gain a better understanding of such information is through the use of the National Institutes of Health’s Genotype-Tissue Expression project (GTEx) database. The GTEx dataset includes genotype, expression, and basic demographic data for nearly 1000 donors of up to 53 different tissues. Within the GTEx database, a web-based portal can be used to check sample and tissue distributions, to browse and search data by various parameters, and to query expression Quantitative Trait Loci (eQTL). While the portal information is useful for general inquiry, raw data access (genotype, expression, and a limited set of demographic and phenotypic data) can be granted for more detailed projects by application through the National Center for Biotechnology Information’s Database of Genotypes and Phenotypes (dbGAP; https://dbgap.ncbi.nlm.nih.gov/). Here, we applied and obtained such access to the controlled access dataset to investigate eQTL in seven dopamine pathway genes (including ankyrin repeat and kinase domain containing 1 [*ANKK1*], dopamine beta-hydroxylase [*DBH*], dopamine receptor D1 [*DRD1*], dopamine receptor D2 [*DRD2*], dopamine receptor D3 [*DRD3*], dopamine receptor D5 [*DRD5*], and dopamine transporter 1/solute carrier family 6 member 3 [*SLC6A3*]) across four human brain tissues, selected for their importance in dopaminergic reward, movement, and memory processes: prefrontal cortex, nucleus accumbens, substantia nigra, and hippocampus. A wide variety of psychiatric and neurological disease processes are associated with these genes and brain regions. In particular, single nucleotide polymorphisms (SNPs) in *ANKK1* and *DRD2* have been associated with addictive behaviors [1,2] and schizophrenia [3,4], while variants in *DBH*, *DRD1*, and *DRD3* have been associated with Alzheimer’s Disease, Parkinson’s Disease, and cognitive performance in a number of different clinical populations [5,6,7]. Additionally, variants in *DRD5* and *SLC6A3* have been associated with attention deficit hyperactivity disorder (ADHD) [8]. The purpose of this work was to investigate genotype by expression changes across these tissues, with the hope that this exploratory inquiry will build a foundation for future work in finding disease risk variants and potential targets for therapeutic intervention. To our knowledge, this work is the first of its kind in summing the pathway effects of genetic variation for expression in dopaminergic genes for human brain tissue samples. 

## 2. Materials and Methods

After receiving access to the controlled dataset and obtaining approval of this protocol under the designation of non-human subject research by the Institutional Review Board of the University of Tennessee Health Science Center (#19-06937-NHSR), our team downloaded expression data (gene read counts or Transcript Per Kilobase Million [TPMs]), sample attributes, and subject demographic and phenotype data from the GTEx portal. The results reported here are based on GTEx data phs000424.GTEx.v8.p2.c1.GRU with serial reformatting, genotype data GTEx_Analysis_2017-06-05_v8_WholeGenomeSeq_838Indiv_Analysis_Freeze.vcf, and expression data phASER_GTEx_v8_matrix.gw_phased.txt from phe000037.v1.GTEx_v8_RNAseq.expression-data-matrixfmt.c1. We are interested in and limited our inquiries to seven dopaminergic genes (*ANKK1*, *DBH*, *DRD1*, *DRD2*, *DRD3*, *DRD5*, and *SLC6A3*) across the following four tissues as described in the database: brain—nucleus accumbens (basal ganglia), brain—substantia nigra, brain—hippocampus, and brain—frontal cortex (BA9; representing prefrontal cortex), with the purpose to identify SNPs associated with corresponding gene expression across the four brain tissues. The genotype variants within 10 kilobases up- and down-stream to the seven genes were extracted and coded as the number of alternative alleles (0, 1, and 2). The total RNAseq expression values of the seven genes in dopamine pathway were also extracted for each of these four tissues. Any expression value of 0 was excluded from any association analysis, as this could mean no expression, or the expression level was under the detection threshold level. The association of each gene expression in a brain tissue was tested using rank-based Kruskal–Wallis test with sex and race. The association of expression with number of alternative alleles of variants annotated to the corresponding gene in dopamine pathway was measured by Spearman correlation, a rank-based conservative approach. The magnitude of the rank-based correlation reflects the degree with which the SNP effects expression between brain tissues. False discovery rate was controlled by Benjamini–Hochberg method, with q value cutoff of 0.2 in each of the 4 brain tissues [9]. As only the variants annotated to the seven genes in dopamine pathway were interrogated, the selection criteria were less stringent than would be required by a whole genome approach. All analyses were performed in R-3.6.1 [10]. 

## 3. Results

### 3.1. Single Tissue Genotype x Expression Analyses

**Figure 1:**
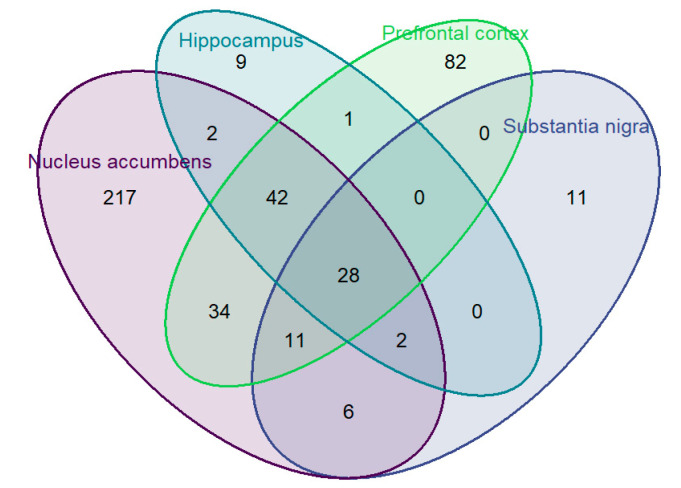
Venn diagram of cis- expression Quantitative Trait Loci (eQTL) of the selected seven genes in the dopamine pathway across four brain tissues.

A total of 6602 variants were annotated to the selected seven genes in the dopamine pathway and within 10 kilobases up- and down-stream of the gene. The variants with minor allele frequency (MAF) less than 0.01 or call rate less than 0.9 were excluded from analysis. In total, 1367 variants were interrogated in the association analysis with corresponding gene expression. Table 1 demonstrates the distribution of significant associations of variants by tissue for each gene at false discovery rate (FDR) of 0.2. Information on the results for each individual variant is provided in Supplementary Table S1. The overlap of significant variants for expression between tissues is demonstrated in the Venn diagram of Figure 1.

### 3.2. Single Tissue Expression Analyses by Demographic and Phenotypic Characteristics

For each individual tissue type, we next investigated general demographic and phenotype characteristics (such as age, sex, race, and body mass index [BMI]) for associations with expression to uncover patterns of variation in gene expression across tissues. The association of each gene with at least 30% non-zero expression in a brain tissue with sex and race was tested using Kruskal–Wallis test. The association with age and BMI was measured by Spearman correlation. A limitation of this dataset is that few subjects had full genetic and RNAseq data for all four tissues, and so sample size and demographic associations with genotype x expression varied between 51 and 177 individuals, for a total of 249 unique persons in the dataset. Demographic characteristics for this group of subjects can be found in Supplementary Table S2, but in brief this group was 71.89% male and 90.73% white, had a median age of 61 years old (range: 55–66 years), and had a median BMI of 27.44 (range: 24.99–31.32). We found that age was inversely associated with expression within nucleus accumbens for *DRD1* (n = 145; r = −0.1977; *p = 0*.017) and *DRD3* (n = 136; r = −0.1879; *p = 0*.029), in hippocampus for *DRD5* (n = 89; r = −0.2648; *p = 0*.012), and in prefrontal cortex for *DRD1* (n = 123; r = −0.2212; *p = 0*.014) and *DRD5* (n = 103; r = −0.3179; *p = 0*.001; these associations and demographic information can be found in Supplementary Table S3). Increased expression was associated with female sex within substantia nigra for *DRD5* (n = 51; *p = 0*.018) and *SLC6A3* (n = 100; *p = 0*.044), but with male sex in hippocampus for *DRD5* (n = 89; *p = 0*.05; Supplementary Table S3). Although race was found to be significantly associated with expression for *DRD3* in nucleus accumbens (*p = 0*.0002), *DRD2* in substantia nigra (*p = 0*.047), and *DBH* in prefrontal cortex (n = 161; *p = 0*.018), the majority (>90%) of each sample was Caucasian, limiting the conclusions that could be drawn from this information. There were particularly limited numbers of samples collected from Asian subjects. Within substantia nigra, expression was associated with BMI for *DRD2* (n = 87; *p = 0*.05) but there were no other associations in other tissues or genes with this characteristic. 

### 3.3. Four Tissue Genotype x Expression Analyses 

Next, we are interested in the SNPs associated with gene expression across all four brain tissues. Out of 1367 variants interrogated, there are 28 variants in three linkage disequilibrium (LD) blocks that were associated with alterations in expression of *DRD2* across all four tissues (see the quad overlapping portion of Venn diagram in Figure 1, LD structure for the three haplotype blocks is presented in Supplementary Figure S1) at FDR of 0.2 cutoff. The first LD block contains intron variant rs7103679 in *DRD2*. The second LD block contains two variants: rs6277 and rs10891549, with correlation of 0.99, and average correlation of −0.42 with the third LD block, which contains 25 variants with smallest correlation of 0.93. Detail on these associations in each tissue is provided in Table 2, with shading indicating respective LD blocks. 

The variants in LD block 1 and 3 are all intronic or fall outside of the coding region of *DRD2*. For rs10891549, the reference allele incorporates a thymine and the alternate allele incorporates a cytosine at chr11:113407725 (GRCh38.p12). The minor allele frequency (MAF) from 1000 genomes estimated ~0.26 for the alternate allele, although the variant is less common in African and Asian populations with MAF < 0.13 (information from dbSNP). In rs6277, a synoynmous variant, the incorporation of the amino acid proline does not change whether the reference allele guanine or alternate allele adenine is present at chr11:113412737 (GRCh38.p12). Similarly to rs10891549, MAF from 1000 genomes is estimated at ~0.24 for the alternate allele, while the variant is less common in African and Asian populations (MAF < 0.07). For full detail on all *DRD2* SNPs in each tissue, please see Supplementary Table S1. As there was no significant association between total RNAseq expressions and sex or race in each tissue, the association tests of expression with variants was not adjusted for demographics or population structure for the target pathway. Detail on the effect of the genotype x expression associations for each variant and tissue in our three LD blocks is detailed in Table 2, whereas a negative correlation indicates that the number of alternate alleles is associated with lower expression of *DRD2* for that tissue (i.e., the number of C alleles in rs7103679 is negatively associated with *DRD2* expression in all four tissues, with the association in hippocampus the strongest correlation (r = −0.339, *p* = 0.0002) and the weakest correlation occurring in prefrontal cortex (r = −0.252, *p* = 0.0048) for these four tissues). 

## 4. Discussion

Our work is some of the first of its kind to investigate dopaminergic genotype by expression changes in human brain tissue samples. Our results identified demographic and phenotypic characteristics associated with alterations in gene expression, and also identified individual variants within each gene and within each tissue that are associated with these expression changes. Such information may provide additional evidence for the association of various disease states with particular demographic and phenotypic characteristics, and may provide a foundation for further inquiry into the circuitry of gene expression alterations in each disease state. Furthermore, our results demonstrate that there are three haplotype blocks, for a total of 28 *DRD2* SNPs that are associated with expression across our four tissues. In regard to demographic and phenotypic characteristics, we found associations of gene expression with age, sex, and BMI. Each is discussed in turn. 

### 4.1. Age

We noted that age inversely alters expression of *DRD1* in the nucleus accumbens and prefrontal cortex, *DRD3* in the nucleus accumbens, and of *DRD5* in the hippocampus and prefrontal cortex. Clinically, such findings hint at the potential for age-related changes in expression to be associated with the development or worsening of neurological and psychiatric conditions; older age is a common risk factor for such changes. However, very little work has been done in human brain tissues to determine the mechanism by which such associations occur. We do know that upregulation of *DRD1*, *DRD3*, and *DRD5* expression in blood, along with alterations in the expression of other dopaminergic signaling genes, has been associated with lower resilience to stressful events and other psychological parameters, including personality, depression, anxiety, and intelligence, in age, sex, and race-matched individuals [11]. We also know that increasing binding of D1 receptors has been associated with younger age in suicide [12], and *DRD5* variants have been associated with age of onset in pediatric attention-deficit/hyperactivity disorder (ADHD) patients [13]. Such information further supports our findings. 

One study investigated expression in *DRD1*, *DRD2*, and *DRD5* (among other dopaminergically relevant genes) in dorsolateral prefrontal cortex samples (Brodmann’s area 46) taken from 69 patients, grouped into seven different age groups (considering categorically neonates, infants, toddlers, young adults, and adults) [14]. The sample from this study was significantly younger than our sample at hand (our sample age range was from 55–66 years at time of death; theirs was from 0.1–50 years old), but found that *DRD1* expression was correlated positively with age, with adults having the highest expression, while *DRD2* and *DRD5* expression levels were inversely correlated with age [14]. While this information is in agreement with our findings on *DRD5* expression, it is in disagreement with our findings for *DRD1*, and *DRD2* expression was not found to be associated with age for our variants of interest in our sample. These discrepancies could be from analyzing our data specifically in relationship to genotypic variation, in treating age as a continuous rather than a categorical variable, or it could be due to the different age ranges of the subjects at time of death. Differences in sampling locations within the prefrontal cortex (Brodmann’s area 9 in our sample) could also contribute to the discrepancies, as these areas may exhibit differential expression patterns. 

In humans, age-related expression associations by tissue are likely to be highly complex and may be due to a complement of environmental factors that occur over the lifespan. More controlled animal models have found some mixed results. Rat models of aging did not show this variation in *DRD1* expression by age, though other genes that contribute to dopamine activity (such as *NURR1*, *GCH1*, *NTRK2*, and *GFRA1*) showed a relationship to aging [15]. Mouse models of aging demonstrate a relationship with *DRD2* expression but not with other dopamine receptors [16]. Future work should include epigenetic analysis to determine the mechanism for expression alteration in aging, work that has already shown promise in relationship to the dopamine transporter (*DAT*) in rat models of aging [17].

### 4.2. Sex

Our results demonstrate that sex was associated with expression of *DRD5*, with higher expression in females in substantia nigra and higher expression in males in the hippocampus. Higher expression of *SLC6A3* was found in females in substantia nigra. This result is interesting, as sex has long been known to be a risk factor for several different neurological and psychiatric disease processes. In particular, males are at higher risk for Parkinson’s disease (PD; many of the symptoms of this disease are associated with reduced dopamine activity in substantia nigra), ADHD (hippocampal volume is reduced in persons with this disorder), and autism (also with hippocampal effects related to long-term memory), all of which may be linked to lower expression of dopaminergically active genes across our tissues of interest [18]. Furthermore, brain morphology and cognitive function is known to be sexually dimorphic in schizophrenia, a condition which is highly associated with dopaminergic genetic variation and expression [19]. We are only just beginning to understand the impact of such biological influences of sex. However, expression of sex chromosome genes, such as sex-determining region on the Y chromosome (*SRY*), may interact with expression of dopaminergic genes to create such differences [20], though this idea should be investigated further. 

### 4.3. BMI

Although obesity is often associated with dopaminergically active genes (such as in binge eating disorder [21]), in this inquiry, BMI was only associated with expression of *DRD2* in our sample within substantia nigra. There were no other associations with this phenotype in other brain tissues or genes studied in this work. Still, our work adds to the growing body of literature on the involvement of *DRD2* in weight and obesity [21,22,23], and is in agreement with PET imaging of obese subjects that demonstrated increased D2 availability in substantia nigra [24]. Work in animal models has demonstrated altered *DRD2* gene expression in animals that were resistant to calorie-restriction weight loss [25]. Further inquiry is needed to uncover the potential of *DRD2* targets for weight loss management. 

### 4.4. Genotype x Expression Associations

Apart from the demographic and phenotypic inquiry, we found that there were three haplotype blocks of SNPs within the *DRD2* gene that were associated with expression across all four brain tissues. In brief, the *DRD2* gene codes for the dopamine receptor type 2, a G-protein-coupled receptor that has a very strong affinity for binding dopamine and functions to inhibit adenylyl cyclase activity. The gene is located on Chromosome 11q23.2, with the genomic coordinates 11:113, 409, 594–113, 475, 397. Variants in this gene are associated with schizophrenia [3,4], and addictive behaviors, including alcoholism and nicotine use [1,2], substance abuse disorder [26,27], and weight/binge eating behavior [21,22,23]. 

The first SNP, rs7103679, is intronic and is not reported to have clinical significance in the literature to date. However, variants in the second and third blocks have been associated with various neurological and psychiatric diseases in the literature. We will discuss these associations by haplotype block in turn. 

### 4.5. Haplotype Block 2: rs10891549 and rs6277

The first variant within this block is rs10891549. This is a silent variation with no amino acid consequence, but still the variant has been associated with alcohol consumption in the literature [28]. No other results have been indexed in PubMed, but this SNP is in high LD with a much more well-studied variant, rs6277. 

The second variant within *DRD2* found to be associated with expression across the four brain tissues of interest is rs6277. This variant (also known as C957T) is more widely reported in the literature. Although the structure of the amino acid chain is not altered, this polymorphism does still alter mRNA expression overall, resulting in altered protein folding, which reduces translation and receptor synthesis [29]. Alone, the variant has been shown to be associated with higher fiber tract integrity for connections from basal ganglia to frontal cortex [30], a fact that may result from altered expression of the alternate allele, and a fact that supports the current findings. However, the variant is known to be in linkage disequilibrium not only with rs10891549, but also with other well-studied variants Taq1A (rs1800497) and -141C ins/del (rs1799732), located in the promoter region of this gene. Indeed, when the haplotype block CCGCCGTT (rs6277-rs1076560-rs2283265-rs2734833-rs2075652-rs1079596-rs4436578-rs11214607) was considered, phenotypic associations of the block were found to be associated with heroin dependence behavior [26]. As a clinically actionable target, a metanalysis has supported the hypothesis that rs6277 potentially alters response to risperidone treatment of schizophrenia [31], possibly doing so through altered expression, although a definitive conclusion could not be found at this time. 

### 4.6. Haplotype Block 3: 25 Variants in DRD2

The 25 variants that make up this haplotype block are shown in Table 2. Several of these have been reported in the literature, either alone or in portions of the block demonstrated here. Overall, variants in this block are associated with alterations in the D2 short/long isoform expression ratio, schizophrenia and schizotypal symptomatology [32], altered fronto-striatal networks [33], responsiveness to neuroactive medications, and substance abuse disorders. To highlight a few associations, rs2283265 has demonstrated a differential effect by female sex for cocaine addiction (along with a 48 bp VNTR), but in mixed-sex groups the variant has been shown to confer risk of cocaine overdose leading to death (OR ~3 [34]). Sex-based effects have also been found for ADHD symptomotolgoy for rs1079727 and rs1124491 [35].

Additionally, within this same block, the rs1076560 T allele has been associated with responsiveness to anti-psychotic medications [36] and reduction in symptomatology in pharmacologic treatment of Parkinson’s Disease [37], but was most recently demonstrated to be associated with the personality characteristics of neuroticism and anxiety in polysubstance dependent males [38]. However, other studies have not found an association for this variant and substance use disorder [39,40]. In conjunction with rs2242593, the two variants together have been shown to result in higher dopamine binding in the ventral striatum for healthy adults; our work here indicates that the phenomenon may continue in other regions of the brain [41]. 

### 4.7. Limitations

Despite these interesting results, we had several limitations in the conduction of this first-of-its-kind inquiry. First, due to the nature of this large-scale but preexisting database of human tissue samples, there were varying sample sizes between tissues and genes. More problematically for clinical implications of this work, detailed phenotypic data were not available for diseases and processes that would be of high interest. Data on subject health histories, underlying diseases/co-morbidities, and medication histories are not available. Considering the prior work in the genetics of our variants of interest, we feel that any information that could have been provided on schizophrenia, Parkinson’s disease, ADHD, drug use, and connectivity (structural integrity) of these regions would greatly enhance the work, but is unfortunately not available through this resource. Educational level would also be of relevant interest for this work but was similarly not included in the dataset. Furthermore, this dataset is racially quite homogenous (>90% Caucasian). Although we had significant results for expression variation by race, such lack of variability is likely to have skewed the result. Racial/ancestral marker associations should be investigated further in other projects. 

### 4.8. Conclusions

Still, despite these limitations, we found that age alters expression of *DRD1* in the nucleus accumbens and prefrontal cortex, *DRD3* in the nucleus accumbens, and *DRD5* in the hippocampus and prefrontal cortex. Sex was associated with expression of *DRD5* in substantia nigra and hippocampus, and *SLC6A3* in substantia nigra. We found that three blocks of SNPs, all located in *DRD2*, were associated with alterations in expression across all four tissues of interest. These demographic characteristics and these variants should be further investigated for use in screening, diagnosis, and future treatment of neurological and psychiatric conditions. 

## Figures and Tables

**Table 1:** Genotype x expression variants by tissue.

Gene	Total Number of Genotype Variants	Number of Variants in Genotype x Expression Analysis	Number of Significant Genotype x Expression Variants in Nucleus Accumbens	Number of Significant Genotype x Expression Variants in Substantia Nigra	Number of Significant Genotype x Expression Variants in Hippocampus	Number of Significant Genotype x Expression Variants in Prefrontal Cortex
*ANKK1*	512	119	37	1	1	27
*DBH*	1011	232	25	14		65
*DRD1*	404	99	14		11	16
*DRD2*	1368	270	113	32	65	69
*DRD3*	1335	299	163	2	2	7
*DRD5*	478	68	2			1
*SLC6A3*	1494	280	7	9	5	13
Total	6602	1367	361 ^1^	58	84	198

^1^ Note: The total number of variants for nucleus accumbens in Figure 1 is 342. Nineteen variants mapped to the overlapping region of ankyrin repeat and kinase domain containing 1 (*ANKK1*) and dopamine receptor D2 (*DRD2*) and are included for both genes in this table, leading to the discrepancy in the total between the figure and table for this tissue.

**Table 2:** Associations of *DRD2* genotype variants with expression, by tissue.

				Nucleus Accumbens		Substantia Nigra		Hippocampus		Prefrontal Cortex	
Rs ID	Ref. Allele	Alt. Allele	Alt. Allele Frequency	r	*p*	r	*p*	r	*p*	r	*p*
rs7103679	T	C	0.849	−0.293	7.42 × 10^−5^	−0.295	0.0056	−0.339	0.0002	−0.252	0.0048
rs10891549	T	C	0.493	−0.321	1.29 × 10^−5^	−0.334	0.0016	−0.305	0.0007	−0.186	0.0390
rs10891549 *	T	C	0.493	−0.182	2.47 × 10^−2^	−0.334	0.0016	−0.305	0.0007	−0.186	0.0390
rs6277	G	A	0.490	−0.316	1.81 × 10^−5^	−0.334	0.0016	−0.293	0.0012	−0.190	0.0349
rs1079596	C	T	0.158	0.300	5.02 × 10^−5^	0.306	0.0039	0.333	0.0002	0.286	0.0013
rs1125394	T	C	0.157	0.296	6.51 × 10^−5^	0.325	0.0023	0.333	0.0002	0.286	0.0013
rs2471857	C	T	0.153	0.295	6.94 × 10^−5^	0.317	0.0028	0.322	0.0004	0.301	0.0007
rs2471854	G	C	0.152	0.287	1.14 × 10^−4^	0.317	0.0028	0.309	0.0007	0.288	0.0012
rs11214599	C	T	0.151	0.285	1.22 × 10^−4^	0.290	0.0064	0.319	0.0004	0.261	0.0035
rs2511520	C	T	0.151	0.285	1.22 × 10^−4^	0.290	0.0064	0.319	0.0004	0.261	0.0035
rs2242591	C	T	0.151	0.285	1.22 × 10^−4^	0.290	0.0064	0.319	0.0004	0.261	0.0035
rs6278	C	A	0.151	0.285	1.22 × 10^−4^	0.290	0.0064	0.319	0.0004	0.261	0.0035
rs1124491	G	A	0.151	0.285	1.22 × 10^−4^	0.290	0.0064	0.319	0.0004	0.261	0.0035
rs1079595	A	C	0.151	0.285	1.22 × 10^−4^	0.290	0.0064	0.319	0.0004	0.261	0.0035
rs1079594	A	C	0.151	0.285	1.22 × 10^−4^	0.290	0.0064	0.319	0.0004	0.261	0.0035
rs1076560	C	A	0.149	0.288	1.02 × 10^−4^	0.290	0.0064	0.325	0.0003	0.263	0.0032
rs2471855	C	G	0.149	0.291	8.48 × 10^−5^	0.278	0.0093	0.316	0.0005	0.305	0.0006
rs2471851	A	C	0.149	0.271	2.68 × 10^−4^	0.276	0.0097	0.316	0.0005	0.279	0.0017
rs55900980	TG	T	0.146	0.296	6.18 × 10^−5^	0.278	0.0093	0.328	0.0003	0.298	0.0008
rs2283265	C	A	0.146	0.296	6.18 × 10^−5^	0.278	0.0093	0.328	0.0003	0.298	0.0008
rs2075654	C	T	0.146	0.296	6.18 × 10^−5^	0.278	0.0093	0.328	0.0003	0.298	0.0008
rs1079727	T	C	0.146	0.296	6.18 × 10^−5^	0.278	0.0093	0.328	0.0003	0.298	0.0008
rs2734836	C	T	0.146	0.296	6.18 × 10^−5^	0.278	0.0093	0.328	0.0003	0.298	0.0008
rs1962262	G	A	0.146	0.296	6.18 × 10^−5^	0.278	0.0093	0.328	0.0003	0.298	0.0008
rs1079598	A	G	0.146	0.296	6.18 × 10^−5^	0.278	0.0093	0.328	0.0003	0.298	0.0008
rs11214601	C	T	0.153	0.304	3.98 × 10^−5^	0.290	0.0064	0.337	0.0002	0.281	0.0016
rs2242593	T	C	0.153	0.304	3.98 × 10^−5^	0.290	0.0064	0.337	0.0002	0.281	0.0016
rs1125393	C	T	0.147	0.292	8.69 × 10^−5^	0.278	0.0093	0.303	0.0009	0.292	0.0010
rs7350522	G	T	0.146	0.271	2.66 × 10^−4^	0.276	0.0097	0.328	0.0003	0.243	0.0065

* This variant is also annotated to ANKK1. The background color clearly shows the three different blocks (block one - rs7103679, block two - rs10894 and rs6277, and block three – SNPs).

## Supplementary Materials

The following are available online at https://www.mdpi.com/2076-3425/10/8/567/s1, Table S1: Genotype x expression variants by tissue (detailed information). Table S2: Demographic and phenotypic characteristics for participants (*N* = 249). Table S3: Association of gene expression with demographic and phenotypic characteristics, by tissue. Figure S1. Linkage disequilibrium structure for 3 haplotype blocks of *DRD2*.

## References

[1] Bühler K.-M., Giné E., Alzate V.E., Calleja-Conde J., De Fonseca F.R., López-Moreno J. (2015). Common single nucleotide variants underlying drug addiction: More than a decade of research. Addict. Biol..

[2] Clarke T.-K., Adams M.J., Davies G., Howard D.M., Hall L.S., Padmanabhan S., Murray A.D., Smith B.H., Campbell A., Hayward C. (2017). Genome-wide association study of alcohol consumption and genetic overlap with other health-related traits in UK Biobank (N=112 117). Mol. Psychiatry.

[3] Howes O.D., McCutcheon R.A., Owen M.J., Murray R.M. (2017). The Role of Genes, Stress, and Dopamine in the Development of Schizophrenia. Biol. Psychiatry.

[4] Nakata Y., Kanahara N., Iyo M. (2017). Dopamine supersensitivity psychosis in schizophrenia: Concepts and implications in clinical practice. J. Psychopharmacol..

[5] Tang S., Yao B., Li N., Lin S., Huang Z. (2018). Association of Dopamine Beta-Hydroxylase Polymorphisms with Alzheimer’s Disease, Parkinson’s Disease and Schizophrenia: Evidence Based on Currently Available Loci. Cell. Physiol. Biochem..

[6] Kang S., Bi M., Du X., Jiao Q., Jiang H. (2018). Association of the rs1611115 polymorphism in DBH gene with Parkinson’s disease: A meta-analysis. Neurol. Sci..

[7] Correa D.D., Satagopan J., Martin A., Braun E., Kryza-Lacombe M., Cheung K., Sharma A., Dimitriadoy S., O’Connell K., Leong S. (2019). Genetic variants and cognitive functions in patients with brain tumors. Neuro-Oncology.

[8] Al-Hawi Z., Cummins T.D.R., Tong J., Johnson B.P., Lau R., Samarrai W., Bellgrove M.A. (2015). The molecular genetic architecture of attention deficit hyperactivity disorder. Mol. Psychiatry.

[9] Benjamini Y., Hochberg Y. (1995). Controlling the False Discovery Rate: A Practical and Powerful Approach to Multiple Testing. J. R. Stat. Soc. Ser. B Stat. Methodol..

[10] R Core Team (2016). R: A Language and Environment for Statistical Computing.

[11] Azadmarzabadi E., Haghighatfard A., Mohammadi A. (2018). Low resilience to stress is associated with candidate gene expression alterations in the dopaminergic signalling pathway. Psychogeriatrics.

[12] Fitzgerald M.L., A Kassir S., Underwood M.D., Bakalian M.J., Mann J.J., Arango V. (2016). Dysregulation of Striatal Dopamine Receptor Binding in Suicide. Neuropsychopharmacology.

[13] Maitra S., Sarkar K., Sinha S., Mukhopadhyay K. (2016). The Dopamine Receptor D5 May Influence Age of Onset. J. Child Neurol..

[14] Rothmond D.A., Weickert C.S., Webster M.J. (2012). Developmental changes in human dopamine neurotransmission: Cortical receptors and terminators. BMC Neurosci..

[15] Parkinson G.M., Dayas C.V., Smith D.W. (2015). Age-related gene expression changes in substantia nigra dopamine neurons of the rat. Mech. Ageing Dev..

[16] Rani M., Kanungo M. (2006). Expression of D2 dopamine receptor in the mouse brain. Biochem. Biophys. Res. Commun..

[17] Green A.L., Eid A., Zhan L., Zarbl H., Guo G.L., Richardson J.R. (2019). Epigenetic Regulation of the Ontogenic Expression of the Dopamine Transporter. Front. Genet..

[18] Pinares-Garcia P., Stratikopoulos M., Zagato A., Loke H., Lee J. (2018). Sex: A Significant Risk Factor for Neurodevelopmental and Neurodegenerative Disorders. Brain Sci..

[19] Mendrek A., Mancini-Marïe A. (2016). Sex/gender differences in the brain and cognition in schizophrenia. Neurosci. Biobehav. Rev..

[20] Loke H., Harley V., Lee J. (2015). Biological factors underlying sex differences in neurological disorders. Int. J. Biochem. Cell Biol..

[21] Sun X., Luquet S.H., Small D.M. (2017). DRD2: Bridging the Genome and Ingestive Behavior. Trends Cogn. Sci..

[22] Blum K., Liu Y., Shriner R., Gold M.S. (2011). Reward circuitry dopaminergic activation regulates food and drug craving behavior. Curr. Pharm. Des..

[23] Stanfill A., Hathaway D., Cashion A., Homayouni R., Cowan P., Thompson C., Madahian B., Conley Y. (2015). A Pilot Study of Demographic and Dopaminergic Genetic Contributions to Weight Change in Kidney Transplant Recipients. PLoS ONE.

[24] Gaiser E.C., Gallezot J.-D., Worhunsky P.D., Jastreboff A.M., Pittman B., Kantrovitz L., Angarita G.A., Cosgrove K.P., Potenza M.N., Malison R.T. (2016). Elevated Dopamine D2/3 Receptor Availability in Obese Individuals: A PET Imaging Study with [11C](+)PHNO. Neuropsychopharmacology.

[25] Vaanholt L.M., Mitchell S.E., Sinclair R.E., Speakman J.R. (2015). Mice that are resistant to diet-induced weight loss have greater food anticipatory activity and altered melanocortin-3 receptor (MC3R) and dopamine receptor 2 (D2) gene expression. Horm. Behav..

[26] Gao X., Wang Y., Lang M., Yuan L., Reece A.S., Wang W. (2017). Contribution of Genetic Polymorphisms and Haplotypes in DRD2, BDNF, and Opioid Receptors to Heroin Dependence and Endophenotypes Among the Han Chinese. OMICS: A J. Integr. Biol..

[27] Zhang J., Yan P., Li Y., Cai X., Yang Z., Miao X., Chen B., Li S.-B., Dang W., Jia W. (2018). A 35.8 kilobases haplotype spanning ANKK1 and DRD2 is associated with heroin dependence in Han Chinese males. Brain Res..

[28] Meyers J.L., Nyman E., Loukola A., Rose R.J., Kaprio J., Dick D.M. (2013). The association between DRD2/ANKK1 and genetically informed measures of alcohol use and problems. Addict Biol..

[29] Duan J., Wainwright M.S., Comeron J.M., Saitou N., Sanders A.R., Gelernter J., Gejman P.V. (2003). Synonymous mutations in the human dopamine receptor D2 (DRD2) affect mRNA stability and synthesis of the receptor. Hum. Mol. Genet..

[30] Markett S., De Reus M.A., Reuter M., Montag C., Weber B., Schoene-Bake J.-C., Heuvel M.P.V.D. (2017). Variation on the dopamine D2 receptor gene (DRD2) is associated with basal ganglia-to-frontal structural connectivity. NeuroImage.

[31] Ma L., Zhang X., Xiang Q., Zhou S., Zhao N., Xie Q., Zhao X., Zhou Y., Cui Y. (2018). Association between dopamine receptor gene polymorphisms and effects of risperidone treatment: A systematic review and meta-analysis. Basic Clin. Pharmacol. Toxicol..

[32] Taurisano P., Romano R., Mancini M., Di Giorgio A., Antonucci L.A., Fazio L., Rampino A., Quarto T., Gelao B., Porcelli A. (2014). Prefronto-striatal physiology is associated with schizotypy and is modulated by a functional variant of DRD2. Front. Behav. Neurosci..

[33] Luykx J.J., Broersen J.L., De Leeuw M. (2017). The DRD2 rs1076560 polymorphism and schizophrenia-related intermediate phenotypes: A systematic review and meta-analysis. Neurosci. Biobehav. Rev..

[34] Stolf A.R., Cupertino R.B., Müller D., Sanvicente-Vieira B., Roman T., Vitola E.S., Grevet E.H., Von Diemen L., Kessler F., Grassi-Oliveira R. (2018). Effects of DRD2 splicing-regulatory polymorphism and DRD4 48 bp VNTR on crack cocaine addiction. J. Neural Transm..

[35] Nyman E.S., Loukola A., Varilo T., Taanila A., Hurtig T., Moilanen I., Loo S., McGough J.J., Järvelin M.-R., Smalley S.L. (2012). Sex-specific influence of DRD2 on ADHD-type temperament in a large population-based birth cohort. Psychiatr. Genet..

[36] Blasi G., Selvaggi P., Fazio L., Antonucci L.A., Taurisano P., Masellis R., Romano R., Mancini M., Zhang F., Caforio G. (2015). Variation in Dopamine D2 and Serotonin 5-HT2A Receptor Genes is Associated with Working Memory Processing and Response to Treatment with Antipsychotics. Neuropsychopharmacology.

[37] Miller N.S., Chou K.L., I Bohnen N., Müller M.L.T.M., Seidler R.D. (2018). Dopaminergic polymorphisms associated with medication responsiveness of gait in Parkinson’s disease. Park. Relat. Disord..

[38] Suchanecka A., Chmielowiec J., Chmielowiec K., Masiak J., Sipak-Szmigiel O., Sznabowicz M., Czarny W., Michałowska-Sawczyn M., Trybek G., Grzywacz A. (2020). Dopamine Receptor DRD2 Gene rs1076560, Personality Traits and Anxiety in the Polysubstance Use Disorder. Brain Sci..

[39] Alblooshi H., Hulse G.K., Osman W., Elkashef A.M., Shawky M., Al Ghaferi H., Alsafar H., Tay G.K. (2018). The frequency of DRD2 rs1076560 and OPRM1 rs1799971 in substance use disorder patients from the United Arab Emirates. Ann. Gen. Psychiatry.

[40] Sznabowicz M., Jasiewicz A., Iskra-Trifunović J., Małecka I., Karakiewicz B., Kotwas A., Samochowiec J., Grzywacz A. (2018). Case-control study analysis of DRD2 gene polymorphisms in drug addicted patients. Psychiatr. Polska.

[41] Valli M., Cho S.S., Masellis M., Chen R., Rusjan P., Kim J., Koshimori Y., Mihaescu A., Strafella A.P. (2019). DRD2 Genotype-Based Variants Modulates D2 Receptor Distribution in Ventral Striatum. Mol. Neurobiol..

