# Supplementary material for: Expression of Dopamine-Related Genes in Four Human Brain Regions
Source: Brain Sci. 2020 Aug 18;10(8):567. doi: 10.3390/brainsci10080567 (PMC7465182; doi:10.3390/brainsci10080567)
Supplement: Supplementary file 1 [file brainsci-10-00567-s001.zip › Supplementary Table 1.docx]

| **Supplementary Table 1.** Genotype x expression variants by tissue (detailed information). | | | | | | | |
| --- | --- | --- | --- | --- | --- | --- | --- |
| **Tissue** | **Gene**  **symbol** | **SNP** | **Alternative Allele Frequency** | **Call Rate** | **Spearman**  **r** | **p-value** | **q-value** |
| Hippocampus | ANKK1 | rs7105965 | 0.024 | 0.994 | 0.235 | 1.06E-02 | 1.65E-01 |
| Hippocampus | DRD1 | rs265977 | 0.143 | 0.994 | 0.443 | 3.11E-06 | 1.96E-04 |
| Hippocampus | DRD1 | rs35916350 | 0.088 | 1.000 | 0.422 | 1.01E-05 | 5.76E-04 |
| Hippocampus | DRD1 | rs28465440 | 0.112 | 1.000 | 0.412 | 1.68E-05 | 7.80E-04 |
| Hippocampus | DRD1 | rs12518222 | 0.098 | 0.994 | 0.410 | 1.86E-05 | 8.59E-04 |
| Hippocampus | DRD1 | rs10463051 | 0.136 | 1.000 | 0.407 | 2.19E-05 | 9.83E-04 |
| Hippocampus | DRD1 | rs5326 | 0.103 | 1.000 | 0.348 | 3.38E-04 | 9.86E-03 |
| Hippocampus | DRD1 | rs265976 | 0.176 | 1.000 | 0.337 | 5.31E-04 | 1.41E-02 |
| Hippocampus | DRD1 | rs267411 | 0.073 | 1.000 | -0.286 | 3.62E-03 | 7.81E-02 |
| Hippocampus | DRD1 | rs265973 | 0.594 | 1.000 | -0.280 | 4.42E-03 | 8.65E-02 |
| Hippocampus | DRD1 | rs265975 | 0.330 | 1.000 | 0.279 | 4.54E-03 | 8.85E-02 |
| Hippocampus | DRD1 | rs265974 | 0.345 | 1.000 | 0.243 | 1.40E-02 | 2.00E-01 |
| Hippocampus | DRD2 | rs7131627 | 0.567 | 0.994 | -0.360 | 6.29E-05 | 2.69E-03 |
| Hippocampus | DRD2 | rs2734832 | 0.567 | 0.994 | -0.355 | 7.86E-05 | 3.32E-03 |
| Hippocampus | DRD2 | rs2734831 | 0.567 | 0.994 | -0.355 | 7.86E-05 | 3.32E-03 |
| Hippocampus | DRD2 | rs1107162 | 0.573 | 0.994 | -0.351 | 9.84E-05 | 4.07E-03 |
| Hippocampus | DRD2 | rs2234690 | 0.573 | 0.994 | -0.351 | 9.84E-05 | 4.07E-03 |
| Hippocampus | DRD2 | rs11608185 | 0.573 | 0.994 | -0.351 | 9.84E-05 | 4.07E-03 |
| Hippocampus | DRD2 | rs7131465 | 0.570 | 0.994 | -0.347 | 1.18E-04 | 4.80E-03 |
| Hippocampus | DRD2 | rs34554234 | 0.561 | 1.000 | -0.344 | 1.30E-04 | 5.25E-03 |
| Hippocampus | DRD2 | rs7103679 | 0.855 | 1.000 | -0.339 | 1.66E-04 | 6.50E-03 |
| Hippocampus | DRD2 | rs11214601 | 0.152 | 1.000 | 0.337 | 1.82E-04 | 6.86E-03 |
| Hippocampus | DRD2 | rs2242593 | 0.152 | 1.000 | 0.337 | 1.82E-04 | 6.86E-03 |
| Hippocampus | DRD2 | rs12798900 | 0.561 | 1.000 | -0.335 | 1.98E-04 | 7.43E-03 |
| Hippocampus | DRD2 | rs12363125 | 0.564 | 1.000 | -0.333 | 2.16E-04 | 7.92E-03 |
| Hippocampus | DRD2 | rs2734835 | 0.564 | 1.000 | -0.333 | 2.16E-04 | 7.92E-03 |
| Hippocampus | DRD2 | rs12800853 | 0.561 | 1.000 | -0.333 | 2.20E-04 | 7.92E-03 |
| Hippocampus | DRD2 | rs1079596 | 0.152 | 1.000 | 0.333 | 2.20E-04 | 7.92E-03 |
| Hippocampus | DRD2 | rs1125394 | 0.152 | 1.000 | 0.333 | 2.20E-04 | 7.92E-03 |
| Hippocampus | DRD2 | rs11608109 | 0.564 | 1.000 | -0.331 | 2.39E-04 | 8.49E-03 |
| Hippocampus | DRD2 | rs7122246 | 0.564 | 1.000 | -0.331 | 2.39E-04 | 8.49E-03 |
| Hippocampus | DRD2 | rs7350522 | 0.139 | 1.000 | 0.328 | 2.70E-04 | 8.71E-03 |
| Hippocampus | DRD2 | rs2734838 | 0.570 | 1.000 | -0.328 | 2.70E-04 | 8.71E-03 |
| Hippocampus | DRD2 | rs1800498 | 0.570 | 1.000 | -0.328 | 2.70E-04 | 8.71E-03 |
| Hippocampus | DRD2 | rs2587548 | 0.570 | 1.000 | -0.328 | 2.70E-04 | 8.71E-03 |
| Hippocampus | DRD2 | rs12808482 | 0.570 | 1.000 | -0.328 | 2.70E-04 | 8.71E-03 |
| Hippocampus | DRD2 | rs1076563 | 0.570 | 1.000 | -0.328 | 2.70E-04 | 8.71E-03 |
| Hippocampus | DRD2 | rs1116313 | 0.570 | 1.000 | -0.328 | 2.70E-04 | 8.71E-03 |
| Hippocampus | DRD2 | rs2734839 | 0.582 | 1.000 | -0.328 | 2.73E-04 | 8.71E-03 |
| Hippocampus | DRD2 | rs2734837 | 0.582 | 1.000 | -0.328 | 2.73E-04 | 8.71E-03 |
| Hippocampus | DRD2 | rs55900980 | 0.142 | 1.000 | 0.328 | 2.74E-04 | 8.71E-03 |
| Hippocampus | DRD2 | rs2283265 | 0.142 | 1.000 | 0.328 | 2.74E-04 | 8.71E-03 |
| Hippocampus | DRD2 | rs2075654 | 0.142 | 1.000 | 0.328 | 2.74E-04 | 8.71E-03 |
| Hippocampus | DRD2 | rs1079727 | 0.142 | 1.000 | 0.328 | 2.74E-04 | 8.71E-03 |
| Hippocampus | DRD2 | rs2734836 | 0.142 | 1.000 | 0.328 | 2.74E-04 | 8.71E-03 |
| Hippocampus | DRD2 | rs1962262 | 0.142 | 1.000 | 0.328 | 2.74E-04 | 8.71E-03 |
| Hippocampus | DRD2 | rs1079598 | 0.142 | 1.000 | 0.328 | 2.74E-04 | 8.71E-03 |
| Hippocampus | DRD2 | rs2734833 | 0.576 | 1.000 | -0.327 | 2.86E-04 | 8.95E-03 |
| Hippocampus | DRD2 | rs1079597 | 0.148 | 1.000 | 0.326 | 2.93E-04 | 9.11E-03 |
| Hippocampus | DRD2 | rs1076560 | 0.145 | 1.000 | 0.325 | 3.13E-04 | 9.68E-03 |
| Hippocampus | DRD2 | rs7131440 | 0.567 | 1.000 | -0.324 | 3.19E-04 | 9.69E-03 |
| Hippocampus | DRD2 | rs7131681 | 0.567 | 1.000 | -0.324 | 3.19E-04 | 9.69E-03 |
| Hippocampus | DRD2 | rs12364051 | 0.570 | 1.000 | -0.323 | 3.45E-04 | 1.00E-02 |
| Hippocampus | DRD2 | rs2471857 | 0.148 | 1.000 | 0.322 | 3.57E-04 | 1.03E-02 |
| Hippocampus | DRD2 | rs11214605 | 0.564 | 1.000 | -0.322 | 3.58E-04 | 1.03E-02 |
| Hippocampus | DRD2 | rs11214599 | 0.148 | 1.000 | 0.319 | 4.02E-04 | 1.12E-02 |
| Hippocampus | DRD2 | rs2511520 | 0.148 | 1.000 | 0.319 | 4.02E-04 | 1.12E-02 |
| Hippocampus | DRD2 | rs2242591 | 0.148 | 1.000 | 0.319 | 4.02E-04 | 1.12E-02 |
| Hippocampus | DRD2 | rs6278 | 0.148 | 1.000 | 0.319 | 4.02E-04 | 1.12E-02 |
| Hippocampus | DRD2 | rs1124491 | 0.148 | 1.000 | 0.319 | 4.02E-04 | 1.12E-02 |
| Hippocampus | DRD2 | rs1079595 | 0.148 | 1.000 | 0.319 | 4.02E-04 | 1.12E-02 |
| Hippocampus | DRD2 | rs1079594 | 0.148 | 1.000 | 0.319 | 4.02E-04 | 1.12E-02 |
| Hippocampus | DRD2 | rs2471851 | 0.142 | 1.000 | 0.316 | 4.67E-04 | 1.26E-02 |
| Hippocampus | DRD2 | rs2471855 | 0.145 | 1.000 | 0.316 | 4.71E-04 | 1.26E-02 |
| Hippocampus | DRD2 | rs11214609 | 0.567 | 1.000 | -0.312 | 5.45E-04 | 1.44E-02 |
| Hippocampus | DRD2 | rs11214608 | 0.564 | 1.000 | -0.310 | 6.04E-04 | 1.57E-02 |
| Hippocampus | DRD2 | rs2471854 | 0.146 | 0.994 | 0.309 | 6.59E-04 | 1.66E-02 |
| Hippocampus | DRD2 | rs4587762 | 0.558 | 1.000 | -0.307 | 6.89E-04 | 1.73E-02 |
| Hippocampus | DRD2 | rs10891549 | 0.473 | 1.000 | -0.305 | 7.41E-04 | 1.85E-02 |
| Hippocampus | DRD2 | rs1554929 | 0.464 | 1.000 | -0.303 | 7.99E-04 | 1.99E-02 |
| Hippocampus | DRD2 | rs1125393 | 0.143 | 0.994 | 0.303 | 8.71E-04 | 2.13E-02 |
| Hippocampus | DRD2 | rs1800497 | 0.218 | 1.000 | 0.299 | 9.58E-04 | 2.30E-02 |
| Hippocampus | DRD2 | rs6277 | 0.470 | 1.000 | -0.293 | 1.21E-03 | 2.84E-02 |
| Hippocampus | DRD2 | rs11214603 | 0.467 | 1.000 | -0.289 | 1.43E-03 | 3.28E-02 |
| Hippocampus | DRD2 | rs11214607 | 0.148 | 1.000 | 0.288 | 1.52E-03 | 3.47E-02 |
| Hippocampus | DRD2 | rs4648319 | 0.148 | 1.000 | 0.288 | 1.52E-03 | 3.47E-02 |
| Hippocampus | DRD2 | rs4938017 | 0.403 | 1.000 | 0.278 | 2.20E-03 | 4.93E-02 |
| Hippocampus | DRD3 | rs11706283 | 0.127 | 1.000 | 0.712 | 4.35E-04 | 1.19E-02 |
| Hippocampus | DRD3 | rs148584133 | 0.106 | 1.000 | 0.610 | 4.29E-03 | 8.42E-02 |
| Hippocampus | SLC6A3 | rs28382245 | 0.403 | 1.000 | 0.449 | 7.73E-03 | 1.36E-01 |
| Hippocampus | SLC6A3 | rs11564758 | 0.403 | 1.000 | 0.449 | 7.73E-03 | 1.36E-01 |
| Hippocampus | SLC6A3 | rs11133778 | 0.399 | 0.994 | 0.449 | 7.73E-03 | 1.36E-01 |
| Hippocampus | SLC6A3 | rs1048955 | 0.409 | 0.994 | 0.449 | 7.73E-03 | 1.36E-01 |
| Hippocampus | SLC6A3 | rs1048953 | 0.406 | 1.000 | 0.449 | 7.73E-03 | 1.36E-01 |
| Nucleus accumbens | ANKK1 | rs4938012 | 0.676 | 1.000 | -0.363 | 4.12E-06 | 1.84E-04 |
| Nucleus accumbens | ANKK1 | rs4938013 | 0.676 | 1.000 | -0.363 | 4.12E-06 | 1.84E-04 |
| Nucleus accumbens | ANKK1 | rs2734847 | 0.802 | 1.000 | -0.348 | 1.03E-05 | 3.15E-04 |
| Nucleus accumbens | ANKK1 | rs2234689 | 0.802 | 1.000 | -0.348 | 1.03E-05 | 3.15E-04 |
| Nucleus accumbens | ANKK1 | rs7945132 | 0.673 | 1.000 | -0.347 | 1.13E-05 | 3.39E-04 |
| Nucleus accumbens | ANKK1 | rs2734848 | 0.772 | 1.000 | -0.336 | 2.19E-05 | 5.39E-04 |
| Nucleus accumbens | ANKK1 | rs17115439 | 0.636 | 1.000 | -0.330 | 3.05E-05 | 7.13E-04 |
| Nucleus accumbens | ANKK1 | rs4938015 | 0.639 | 0.995 | -0.330 | 3.05E-05 | 7.13E-04 |
| Nucleus accumbens | ANKK1 | rs10891546 | 0.636 | 1.000 | -0.330 | 3.05E-05 | 7.13E-04 |
| Nucleus accumbens | ANKK1 | rs877138 | 0.678 | 1.000 | -0.326 | 4.01E-05 | 8.95E-04 |
| Nucleus accumbens | ANKK1 | rs7123797 | 0.639 | 0.995 | -0.326 | 4.09E-05 | 9.10E-04 |
| Nucleus accumbens | ANKK1 | rs3897584 | 0.679 | 0.995 | -0.324 | 4.80E-05 | 1.05E-03 |
| Nucleus accumbens | ANKK1 | rs4601821 | 0.676 | 1.000 | -0.311 | 9.06E-05 | 1.70E-03 |
| Nucleus accumbens | ANKK1 | rs10891543 | 0.676 | 1.000 | -0.311 | 9.06E-05 | 1.70E-03 |
| Nucleus accumbens | ANKK1 | rs12422191 | 0.087 | 1.000 | 0.272 | 6.68E-04 | 8.89E-03 |
| Nucleus accumbens | ANKK1 | rs4938016 | 0.653 | 0.990 | -0.223 | 5.87E-03 | 4.90E-02 |
| Nucleus accumbens | ANKK1 | rs7122228 | 0.656 | 1.000 | -0.221 | 5.96E-03 | 4.93E-02 |
| Nucleus accumbens | ANKK1 | rs11214598 | 0.619 | 0.995 | -0.211 | 9.24E-03 | 6.80E-02 |
| Nucleus accumbens | ANKK1 | rs2587552 | 0.671 | 1.000 | -0.190 | 1.84E-02 | 1.10E-01 |
| Nucleus accumbens | ANKK1 | rs56846012 | 0.648 | 0.990 | -0.189 | 2.02E-02 | 1.13E-01 |
| Nucleus accumbens | ANKK1 | rs2587550 | 0.647 | 0.995 | -0.188 | 2.07E-02 | 1.16E-01 |
| Nucleus accumbens | ANKK1 | rs11283658 | 0.512 | 1.000 | -0.183 | 2.36E-02 | 1.28E-01 |
| Nucleus accumbens | ANKK1 | rs10891549 | 0.493 | 1.000 | -0.182 | 2.47E-02 | 1.32E-01 |
| Nucleus accumbens | ANKK1 | rs34983219 | 0.027 | 1.000 | -0.179 | 2.72E-02 | 1.43E-01 |
| Nucleus accumbens | ANKK1 | rs45542442 | 0.644 | 0.995 | -0.179 | 2.75E-02 | 1.44E-01 |
| Nucleus accumbens | ANKK1 | rs2859544 | 0.644 | 1.000 | -0.177 | 2.82E-02 | 1.46E-01 |
| Nucleus accumbens | ANKK1 | rs1003641 | 0.668 | 1.000 | -0.177 | 2.83E-02 | 1.46E-01 |
| Nucleus accumbens | ANKK1 | rs2859543 | 0.668 | 1.000 | -0.177 | 2.83E-02 | 1.46E-01 |
| Nucleus accumbens | ANKK1 | rs2242592 | 0.667 | 0.995 | -0.178 | 2.87E-02 | 1.47E-01 |
| Nucleus accumbens | ANKK1 | rs2859545 | 0.668 | 1.000 | -0.177 | 2.91E-02 | 1.48E-01 |
| Nucleus accumbens | ANKK1 | rs12360992 | 0.473 | 1.000 | -0.175 | 3.05E-02 | 1.53E-01 |
| Nucleus accumbens | ANKK1 | rs1554929 | 0.488 | 1.000 | -0.173 | 3.25E-02 | 1.62E-01 |
| Nucleus accumbens | ANKK1 | rs34298987 | 0.040 | 1.000 | -0.173 | 3.27E-02 | 1.62E-01 |
| Nucleus accumbens | ANKK1 | rs11214603 | 0.490 | 1.000 | -0.166 | 4.09E-02 | 1.90E-01 |
| Nucleus accumbens | ANKK1 | rs11214602 | 0.641 | 1.000 | -0.164 | 4.22E-02 | 1.93E-01 |
| Nucleus accumbens | ANKK1 | rs2587543 | 0.641 | 1.000 | -0.164 | 4.22E-02 | 1.93E-01 |
| Nucleus accumbens | ANKK1 | rs2734842 | 0.641 | 1.000 | -0.164 | 4.22E-02 | 1.93E-01 |
| Nucleus accumbens | DBH | rs129922 | 0.752 | 1.000 | 0.233 | 2.27E-03 | 2.49E-02 |
| Nucleus accumbens | DBH | rs113249250 | 0.035 | 1.000 | -0.231 | 2.46E-03 | 2.70E-02 |
| Nucleus accumbens | DBH | rs2097629 | 0.418 | 1.000 | 0.224 | 3.27E-03 | 3.36E-02 |
| Nucleus accumbens | DBH | rs2073833 | 0.483 | 1.000 | 0.224 | 3.30E-03 | 3.40E-02 |
| Nucleus accumbens | DBH | rs62575403 | 0.030 | 1.000 | -0.210 | 5.95E-03 | 4.93E-02 |
| Nucleus accumbens | DBH | rs9657707 | 0.035 | 1.000 | 0.207 | 6.79E-03 | 5.43E-02 |
| Nucleus accumbens | DBH | rs7855248 | 0.433 | 1.000 | 0.203 | 8.07E-03 | 6.17E-02 |
| Nucleus accumbens | DBH | rs2097628 | 0.428 | 1.000 | 0.198 | 9.75E-03 | 7.11E-02 |
| Nucleus accumbens | DBH | rs7871224 | 0.433 | 1.000 | 0.197 | 1.02E-02 | 7.35E-02 |
| Nucleus accumbens | DBH | rs7035577 | 0.030 | 1.000 | 0.191 | 1.27E-02 | 8.30E-02 |
| Nucleus accumbens | DBH | rs10761412 | 0.450 | 1.000 | 0.189 | 1.37E-02 | 8.73E-02 |
| Nucleus accumbens | DBH | rs732833 | 0.488 | 1.000 | 0.186 | 1.49E-02 | 9.40E-02 |
| Nucleus accumbens | DBH | rs129883 | 0.755 | 1.000 | 0.183 | 1.68E-02 | 1.03E-01 |
| Nucleus accumbens | DBH | rs2073837 | 0.351 | 1.000 | 0.183 | 1.71E-02 | 1.04E-01 |
| Nucleus accumbens | DBH | rs7851898 | 0.303 | 0.990 | 0.183 | 1.75E-02 | 1.06E-01 |
| Nucleus accumbens | DBH | rs2073836 | 0.371 | 0.995 | 0.182 | 1.81E-02 | 1.08E-01 |
| Nucleus accumbens | DBH | rs199860702 | 0.032 | 1.000 | -0.177 | 2.10E-02 | 1.17E-01 |
| Nucleus accumbens | DBH | rs7854480 | 0.611 | 1.000 | 0.175 | 2.25E-02 | 1.23E-01 |
| Nucleus accumbens | DBH | rs3025419 | 0.027 | 1.000 | 0.174 | 2.36E-02 | 1.28E-01 |
| Nucleus accumbens | DBH | rs73559959 | 0.027 | 1.000 | 0.174 | 2.36E-02 | 1.28E-01 |
| Nucleus accumbens | DBH | rs7876027 | 0.037 | 1.000 | 0.171 | 2.54E-02 | 1.36E-01 |
| Nucleus accumbens | DBH | rs7862335 | 0.544 | 0.901 | 0.175 | 2.87E-02 | 1.47E-01 |
| Nucleus accumbens | DBH | rs73662170 | 0.032 | 1.000 | 0.158 | 3.97E-02 | 1.87E-01 |
| Nucleus accumbens | DBH | rs7862391 | 0.032 | 1.000 | 0.158 | 3.97E-02 | 1.87E-01 |
| Nucleus accumbens | DBH | rs129927 | 0.358 | 0.995 | 0.156 | 4.34E-02 | 1.96E-01 |
| Nucleus accumbens | DRD1 | rs35916350 | 0.094 | 1.000 | 0.335 | 3.91E-05 | 8.86E-04 |
| Nucleus accumbens | DRD1 | rs12518222 | 0.109 | 0.995 | 0.293 | 3.62E-04 | 5.30E-03 |
| Nucleus accumbens | DRD1 | rs5326 | 0.116 | 1.000 | 0.285 | 5.12E-04 | 7.01E-03 |
| Nucleus accumbens | DRD1 | rs28465440 | 0.124 | 1.000 | 0.275 | 8.25E-04 | 1.05E-02 |
| Nucleus accumbens | DRD1 | rs703748 | 0.344 | 1.000 | -0.268 | 1.11E-03 | 1.35E-02 |
| Nucleus accumbens | DRD1 | rs251937 | 0.358 | 0.990 | -0.255 | 2.12E-03 | 2.35E-02 |
| Nucleus accumbens | DRD1 | rs265976 | 0.191 | 1.000 | 0.234 | 4.58E-03 | 4.42E-02 |
| Nucleus accumbens | DRD1 | rs4867798 | 0.279 | 0.995 | 0.223 | 7.13E-03 | 5.60E-02 |
| Nucleus accumbens | DRD1 | rs265977 | 0.162 | 0.995 | 0.209 | 1.17E-02 | 8.09E-02 |
| Nucleus accumbens | DRD1 | rs265975 | 0.334 | 1.000 | 0.209 | 1.18E-02 | 8.09E-02 |
| Nucleus accumbens | DRD1 | rs185482023 | 0.015 | 1.000 | -0.202 | 1.47E-02 | 9.29E-02 |
| Nucleus accumbens | DRD1 | rs835540 | 0.299 | 0.995 | -0.197 | 1.79E-02 | 1.07E-01 |
| Nucleus accumbens | DRD1 | rs265974 | 0.359 | 1.000 | 0.192 | 2.08E-02 | 1.16E-01 |
| Nucleus accumbens | DRD1 | rs10463051 | 0.158 | 1.000 | 0.183 | 2.72E-02 | 1.43E-01 |
| Nucleus accumbens | DRD2 | rs10891549 | 0.493 | 1.000 | -0.321 | 1.29E-05 | 3.83E-04 |
| Nucleus accumbens | DRD2 | rs11214603 | 0.490 | 1.000 | -0.316 | 1.81E-05 | 5.04E-04 |
| Nucleus accumbens | DRD2 | rs6277 | 0.490 | 1.000 | -0.316 | 1.81E-05 | 5.04E-04 |
| Nucleus accumbens | DRD2 | rs1554929 | 0.488 | 1.000 | -0.315 | 1.98E-05 | 5.07E-04 |
| Nucleus accumbens | DRD2 | rs11214601 | 0.153 | 1.000 | 0.304 | 3.98E-05 | 8.95E-04 |
| Nucleus accumbens | DRD2 | rs2242593 | 0.153 | 1.000 | 0.304 | 3.98E-05 | 8.95E-04 |
| Nucleus accumbens | DRD2 | rs1079596 | 0.158 | 1.000 | 0.300 | 5.02E-05 | 1.09E-03 |
| Nucleus accumbens | DRD2 | rs55900980 | 0.146 | 1.000 | 0.296 | 6.18E-05 | 1.25E-03 |
| Nucleus accumbens | DRD2 | rs2283265 | 0.146 | 1.000 | 0.296 | 6.18E-05 | 1.25E-03 |
| Nucleus accumbens | DRD2 | rs2075654 | 0.146 | 1.000 | 0.296 | 6.18E-05 | 1.25E-03 |
| Nucleus accumbens | DRD2 | rs1079727 | 0.146 | 1.000 | 0.296 | 6.18E-05 | 1.25E-03 |
| Nucleus accumbens | DRD2 | rs2734836 | 0.146 | 1.000 | 0.296 | 6.18E-05 | 1.25E-03 |
| Nucleus accumbens | DRD2 | rs1962262 | 0.146 | 1.000 | 0.296 | 6.18E-05 | 1.25E-03 |
| Nucleus accumbens | DRD2 | rs1079598 | 0.146 | 1.000 | 0.296 | 6.18E-05 | 1.25E-03 |
| Nucleus accumbens | DRD2 | rs1079597 | 0.153 | 1.000 | 0.296 | 6.20E-05 | 1.25E-03 |
| Nucleus accumbens | DRD2 | rs1125394 | 0.157 | 0.995 | 0.296 | 6.51E-05 | 1.31E-03 |
| Nucleus accumbens | DRD2 | rs2471857 | 0.153 | 1.000 | 0.295 | 6.94E-05 | 1.37E-03 |
| Nucleus accumbens | DRD2 | rs7103679 | 0.849 | 1.000 | -0.293 | 7.42E-05 | 1.44E-03 |
| Nucleus accumbens | DRD2 | rs7131627 | 0.565 | 0.995 | -0.293 | 8.10E-05 | 1.55E-03 |
| Nucleus accumbens | DRD2 | rs2471855 | 0.149 | 1.000 | 0.291 | 8.48E-05 | 1.62E-03 |
| Nucleus accumbens | DRD2 | rs7131465 | 0.567 | 0.995 | -0.292 | 8.51E-05 | 1.62E-03 |
| Nucleus accumbens | DRD2 | rs1125393 | 0.147 | 0.995 | 0.292 | 8.69E-05 | 1.65E-03 |
| Nucleus accumbens | DRD2 | rs1076560 | 0.149 | 1.000 | 0.288 | 1.02E-04 | 1.89E-03 |
| Nucleus accumbens | DRD2 | rs2471854 | 0.152 | 0.995 | 0.287 | 1.14E-04 | 2.03E-03 |
| Nucleus accumbens | DRD2 | rs11214599 | 0.151 | 1.000 | 0.285 | 1.22E-04 | 2.13E-03 |
| Nucleus accumbens | DRD2 | rs2511520 | 0.151 | 1.000 | 0.285 | 1.22E-04 | 2.13E-03 |
| Nucleus accumbens | DRD2 | rs2242591 | 0.151 | 1.000 | 0.285 | 1.22E-04 | 2.13E-03 |
| Nucleus accumbens | DRD2 | rs6278 | 0.151 | 1.000 | 0.285 | 1.22E-04 | 2.13E-03 |
| Nucleus accumbens | DRD2 | rs1124491 | 0.151 | 1.000 | 0.285 | 1.22E-04 | 2.13E-03 |
| Nucleus accumbens | DRD2 | rs1079595 | 0.151 | 1.000 | 0.285 | 1.22E-04 | 2.13E-03 |
| Nucleus accumbens | DRD2 | rs1079594 | 0.151 | 1.000 | 0.285 | 1.22E-04 | 2.13E-03 |
| Nucleus accumbens | DRD2 | rs2734832 | 0.562 | 0.995 | -0.278 | 1.93E-04 | 3.13E-03 |
| Nucleus accumbens | DRD2 | rs2734831 | 0.562 | 0.995 | -0.278 | 1.93E-04 | 3.13E-03 |
| Nucleus accumbens | DRD2 | rs2242592 | 0.667 | 0.995 | -0.277 | 1.95E-04 | 3.16E-03 |
| Nucleus accumbens | DRD2 | rs7131440 | 0.564 | 1.000 | -0.275 | 2.18E-04 | 3.43E-03 |
| Nucleus accumbens | DRD2 | rs7131681 | 0.564 | 1.000 | -0.275 | 2.18E-04 | 3.43E-03 |
| Nucleus accumbens | DRD2 | rs7350522 | 0.146 | 1.000 | 0.271 | 2.66E-04 | 4.07E-03 |
| Nucleus accumbens | DRD2 | rs2471851 | 0.149 | 1.000 | 0.271 | 2.68E-04 | 4.09E-03 |
| Nucleus accumbens | DRD2 | rs1107162 | 0.565 | 0.995 | -0.269 | 3.04E-04 | 4.61E-03 |
| Nucleus accumbens | DRD2 | rs2234690 | 0.565 | 0.995 | -0.269 | 3.04E-04 | 4.61E-03 |
| Nucleus accumbens | DRD2 | rs11608185 | 0.565 | 0.995 | -0.269 | 3.04E-04 | 4.61E-03 |
| Nucleus accumbens | DRD2 | rs12798900 | 0.557 | 1.000 | -0.266 | 3.41E-04 | 5.09E-03 |
| Nucleus accumbens | DRD2 | rs11214605 | 0.559 | 1.000 | -0.265 | 3.61E-04 | 5.30E-03 |
| Nucleus accumbens | DRD2 | rs12800853 | 0.557 | 1.000 | -0.264 | 3.82E-04 | 5.58E-03 |
| Nucleus accumbens | DRD2 | rs12364051 | 0.564 | 1.000 | -0.261 | 4.50E-04 | 6.38E-03 |
| Nucleus accumbens | DRD2 | rs12363125 | 0.559 | 1.000 | -0.261 | 4.59E-04 | 6.40E-03 |
| Nucleus accumbens | DRD2 | rs2734835 | 0.559 | 1.000 | -0.261 | 4.59E-04 | 6.40E-03 |
| Nucleus accumbens | DRD2 | rs34554234 | 0.557 | 1.000 | -0.260 | 4.85E-04 | 6.70E-03 |
| Nucleus accumbens | DRD2 | rs11608109 | 0.559 | 1.000 | -0.259 | 5.12E-04 | 7.01E-03 |
| Nucleus accumbens | DRD2 | rs7122246 | 0.559 | 1.000 | -0.259 | 5.12E-04 | 7.01E-03 |
| Nucleus accumbens | DRD2 | rs2734839 | 0.579 | 1.000 | -0.258 | 5.21E-04 | 7.10E-03 |
| Nucleus accumbens | DRD2 | rs2734837 | 0.579 | 1.000 | -0.258 | 5.21E-04 | 7.10E-03 |
| Nucleus accumbens | DRD2 | rs4587762 | 0.559 | 1.000 | -0.254 | 6.48E-04 | 8.65E-03 |
| Nucleus accumbens | DRD2 | rs2734838 | 0.562 | 1.000 | -0.252 | 7.08E-04 | 9.26E-03 |
| Nucleus accumbens | DRD2 | rs1800498 | 0.562 | 1.000 | -0.252 | 7.08E-04 | 9.26E-03 |
| Nucleus accumbens | DRD2 | rs2587548 | 0.562 | 1.000 | -0.252 | 7.08E-04 | 9.26E-03 |
| Nucleus accumbens | DRD2 | rs12808482 | 0.562 | 1.000 | -0.252 | 7.08E-04 | 9.26E-03 |
| Nucleus accumbens | DRD2 | rs1076563 | 0.562 | 1.000 | -0.252 | 7.08E-04 | 9.26E-03 |
| Nucleus accumbens | DRD2 | rs1116313 | 0.562 | 1.000 | -0.252 | 7.08E-04 | 9.26E-03 |
| Nucleus accumbens | DRD2 | rs2734833 | 0.569 | 1.000 | -0.252 | 7.10E-04 | 9.26E-03 |
| Nucleus accumbens | DRD2 | rs1124493 | 0.663 | 1.000 | -0.248 | 8.56E-04 | 1.08E-02 |
| Nucleus accumbens | DRD2 | rs6275 | 0.663 | 1.000 | -0.248 | 8.56E-04 | 1.08E-02 |
| Nucleus accumbens | DRD2 | rs2859545 | 0.668 | 1.000 | -0.248 | 8.57E-04 | 1.08E-02 |
| Nucleus accumbens | DRD2 | rs4938017 | 0.408 | 1.000 | 0.248 | 8.82E-04 | 1.10E-02 |
| Nucleus accumbens | DRD2 | rs6279 | 0.661 | 1.000 | -0.244 | 1.07E-03 | 1.31E-02 |
| Nucleus accumbens | DRD2 | rs2587550 | 0.647 | 0.995 | -0.242 | 1.19E-03 | 1.43E-02 |
| Nucleus accumbens | DRD2 | rs56846012 | 0.648 | 0.990 | -0.241 | 1.29E-03 | 1.54E-02 |
| Nucleus accumbens | DRD2 | rs11214602 | 0.641 | 1.000 | -0.239 | 1.36E-03 | 1.61E-02 |
| Nucleus accumbens | DRD2 | rs2587543 | 0.641 | 1.000 | -0.239 | 1.36E-03 | 1.61E-02 |
| Nucleus accumbens | DRD2 | rs2734842 | 0.641 | 1.000 | -0.239 | 1.36E-03 | 1.61E-02 |
| Nucleus accumbens | DRD2 | rs6276 | 0.641 | 1.000 | -0.239 | 1.36E-03 | 1.61E-02 |
| Nucleus accumbens | DRD2 | rs2734841 | 0.666 | 1.000 | -0.235 | 1.65E-03 | 1.93E-02 |
| Nucleus accumbens | DRD2 | rs11214609 | 0.569 | 1.000 | -0.235 | 1.66E-03 | 1.94E-02 |
| Nucleus accumbens | DRD2 | rs11214608 | 0.567 | 1.000 | -0.234 | 1.74E-03 | 2.03E-02 |
| Nucleus accumbens | DRD2 | rs2587552 | 0.671 | 1.000 | -0.233 | 1.83E-03 | 2.13E-02 |
| Nucleus accumbens | DRD2 | rs11214607 | 0.141 | 1.000 | 0.232 | 1.93E-03 | 2.22E-02 |
| Nucleus accumbens | DRD2 | rs4648319 | 0.141 | 1.000 | 0.232 | 1.93E-03 | 2.22E-02 |
| Nucleus accumbens | DRD2 | rs2734847 | 0.802 | 1.000 | -0.230 | 2.11E-03 | 2.35E-02 |
| Nucleus accumbens | DRD2 | rs2234689 | 0.802 | 1.000 | -0.230 | 2.11E-03 | 2.35E-02 |
| Nucleus accumbens | DRD2 | rs1003641 | 0.668 | 1.000 | -0.228 | 2.24E-03 | 2.47E-02 |
| Nucleus accumbens | DRD2 | rs2859543 | 0.668 | 1.000 | -0.228 | 2.24E-03 | 2.47E-02 |
| Nucleus accumbens | DRD2 | rs2859544 | 0.644 | 1.000 | -0.224 | 2.70E-03 | 2.85E-02 |
| Nucleus accumbens | DRD2 | rs45542442 | 0.644 | 0.995 | -0.223 | 2.91E-03 | 3.05E-02 |
| Nucleus accumbens | DRD2 | rs2734848 | 0.772 | 1.000 | -0.218 | 3.54E-03 | 3.60E-02 |
| Nucleus accumbens | DRD2 | rs1800497 | 0.215 | 1.000 | 0.208 | 5.52E-03 | 4.82E-02 |
| Nucleus accumbens | DRD2 | rs12422191 | 0.087 | 1.000 | 0.204 | 6.40E-03 | 5.20E-02 |
| Nucleus accumbens | DRD2 | rs12421616 | 0.054 | 1.000 | -0.196 | 9.06E-03 | 6.69E-02 |
| Nucleus accumbens | DRD2 | rs2471850 | 0.713 | 1.000 | -0.188 | 1.22E-02 | 8.09E-02 |
| Nucleus accumbens | DRD2 | rs2471856 | 0.715 | 1.000 | -0.183 | 1.46E-02 | 9.23E-02 |
| Nucleus accumbens | DRD2 | rs2245805 | 0.729 | 0.995 | -0.181 | 1.62E-02 | 9.97E-02 |
| Nucleus accumbens | DRD2 | rs2511521 | 0.709 | 0.995 | -0.181 | 1.65E-02 | 1.01E-01 |
| Nucleus accumbens | DRD2 | rs4586205 | 0.708 | 1.000 | -0.179 | 1.72E-02 | 1.05E-01 |
| Nucleus accumbens | DRD2 | rs148313257 | 0.012 | 1.000 | -0.179 | 1.72E-02 | 1.05E-01 |
| Nucleus accumbens | DRD2 | rs117090941 | 0.022 | 1.000 | 0.178 | 1.80E-02 | 1.08E-01 |
| Nucleus accumbens | DRD2 | rs10891552 | 0.059 | 1.000 | -0.175 | 1.99E-02 | 1.12E-01 |
| Nucleus accumbens | DRD2 | rs55887984 | 0.059 | 1.000 | -0.175 | 1.99E-02 | 1.12E-01 |
| Nucleus accumbens | DRD2 | rs4648318 | 0.257 | 1.000 | 0.173 | 2.10E-02 | 1.17E-01 |
| Nucleus accumbens | DRD2 | rs11214611 | 0.168 | 1.000 | -0.171 | 2.33E-02 | 1.26E-01 |
| Nucleus accumbens | DRD2 | rs4648317 | 0.168 | 1.000 | -0.171 | 2.33E-02 | 1.26E-01 |
| Nucleus accumbens | DRD2 | rs4245149 | 0.173 | 1.000 | -0.168 | 2.58E-02 | 1.37E-01 |
| Nucleus accumbens | DRD2 | rs10789944 | 0.173 | 1.000 | -0.168 | 2.58E-02 | 1.37E-01 |
| Nucleus accumbens | DRD2 | rs10891551 | 0.166 | 1.000 | -0.167 | 2.61E-02 | 1.38E-01 |
| Nucleus accumbens | DRD2 | rs7117915 | 0.171 | 1.000 | -0.164 | 2.89E-02 | 1.48E-01 |
| Nucleus accumbens | DRD2 | rs4350392 | 0.171 | 1.000 | -0.164 | 2.89E-02 | 1.48E-01 |
| Nucleus accumbens | DRD2 | rs4938019 | 0.171 | 1.000 | -0.164 | 2.89E-02 | 1.48E-01 |
| Nucleus accumbens | DRD2 | rs10789943 | 0.171 | 1.000 | -0.164 | 2.89E-02 | 1.48E-01 |
| Nucleus accumbens | DRD2 | rs10891550 | 0.167 | 0.995 | -0.164 | 2.92E-02 | 1.48E-01 |
| Nucleus accumbens | DRD2 | rs2002453 | 0.725 | 1.000 | -0.163 | 3.07E-02 | 1.54E-01 |
| Nucleus accumbens | DRD2 | rs2005313 | 0.725 | 1.000 | -0.163 | 3.07E-02 | 1.54E-01 |
| Nucleus accumbens | DRD2 | rs1076562 | 0.725 | 1.000 | -0.163 | 3.07E-02 | 1.54E-01 |
| Nucleus accumbens | DRD2 | rs7122454 | 0.168 | 0.990 | -0.163 | 3.17E-02 | 1.58E-01 |
| Nucleus accumbens | DRD2 | rs12574471 | 0.119 | 1.000 | 0.156 | 3.77E-02 | 1.81E-01 |
| Nucleus accumbens | DRD2 | rs72997602 | 0.037 | 1.000 | -0.154 | 4.09E-02 | 1.90E-01 |
| Nucleus accumbens | DRD3 | rs167771 | 0.797 | 1.000 | -0.673 | 2.81E-19 | 6.93E-16 |
| Nucleus accumbens | DRD3 | rs2971569 | 0.617 | 0.995 | -0.623 | 7.00E-16 | 4.84E-13 |
| Nucleus accumbens | DRD3 | rs717668 | 0.616 | 1.000 | -0.621 | 7.85E-16 | 4.84E-13 |
| Nucleus accumbens | DRD3 | rs324035 | 0.785 | 1.000 | -0.620 | 8.90E-16 | 4.88E-13 |
| Nucleus accumbens | DRD3 | rs7638876 | 0.376 | 1.000 | 0.594 | 2.40E-14 | 1.18E-11 |
| Nucleus accumbens | DRD3 | rs1503670 | 0.418 | 0.995 | 0.586 | 8.17E-14 | 3.67E-11 |
| Nucleus accumbens | DRD3 | rs1394016 | 0.418 | 1.000 | 0.582 | 1.14E-13 | 4.71E-11 |
| Nucleus accumbens | DRD3 | rs6280 | 0.641 | 1.000 | -0.564 | 8.87E-13 | 3.24E-10 |
| Nucleus accumbens | DRD3 | rs324026 | 0.641 | 1.000 | -0.564 | 8.87E-13 | 3.24E-10 |
| Nucleus accumbens | DRD3 | rs7638961 | 0.358 | 0.995 | 0.564 | 1.12E-12 | 3.68E-10 |
| Nucleus accumbens | DRD3 | rs324028 | 0.658 | 1.000 | -0.557 | 1.92E-12 | 5.94E-10 |
| Nucleus accumbens | DRD3 | rs7639152 | 0.339 | 1.000 | 0.543 | 8.17E-12 | 2.37E-09 |
| Nucleus accumbens | DRD3 | rs79913906 | 0.673 | 1.000 | -0.481 | 2.97E-09 | 5.23E-07 |
| Nucleus accumbens | DRD3 | rs226082 | 0.673 | 1.000 | -0.481 | 2.97E-09 | 5.23E-07 |
| Nucleus accumbens | DRD3 | rs324030 | 0.673 | 1.000 | -0.481 | 2.97E-09 | 5.23E-07 |
| Nucleus accumbens | DRD3 | rs324029 | 0.673 | 1.000 | -0.481 | 2.97E-09 | 5.23E-07 |
| Nucleus accumbens | DRD3 | rs324023 | 0.673 | 1.000 | -0.481 | 2.97E-09 | 5.23E-07 |
| Nucleus accumbens | DRD3 | rs324022 | 0.675 | 0.990 | -0.482 | 3.72E-09 | 5.82E-07 |
| Nucleus accumbens | DRD3 | rs2971209 | 0.674 | 0.995 | -0.480 | 3.89E-09 | 5.82E-07 |
| Nucleus accumbens | DRD3 | rs324033 | 0.856 | 1.000 | -0.478 | 3.89E-09 | 5.82E-07 |
| Nucleus accumbens | DRD3 | rs11721264 | 0.315 | 0.990 | 0.440 | 1.07E-07 | 1.21E-05 |
| Nucleus accumbens | DRD3 | rs167770 | 0.683 | 1.000 | -0.434 | 1.33E-07 | 1.45E-05 |
| Nucleus accumbens | DRD3 | rs324036 | 0.847 | 1.000 | -0.425 | 2.57E-07 | 2.73E-05 |
| Nucleus accumbens | DRD3 | rs2630347 | 0.847 | 1.000 | -0.425 | 2.57E-07 | 2.73E-05 |
| Nucleus accumbens | DRD3 | rs3773678 | 0.848 | 0.995 | -0.422 | 3.40E-07 | 3.29E-05 |
| Nucleus accumbens | DRD3 | rs2630349 | 0.921 | 1.000 | -0.415 | 5.13E-07 | 4.87E-05 |
| Nucleus accumbens | DRD3 | rs324032 | 0.921 | 1.000 | -0.404 | 1.06E-06 | 8.89E-05 |
| Nucleus accumbens | DRD3 | rs324031 | 0.921 | 1.000 | -0.404 | 1.06E-06 | 8.89E-05 |
| Nucleus accumbens | DRD3 | rs7629232 | 0.364 | 1.000 | 0.394 | 2.09E-06 | 1.12E-04 |
| Nucleus accumbens | DRD3 | rs9880494 | 0.361 | 1.000 | 0.394 | 2.09E-06 | 1.12E-04 |
| Nucleus accumbens | DRD3 | rs6801068 | 0.361 | 1.000 | 0.394 | 2.09E-06 | 1.12E-04 |
| Nucleus accumbens | DRD3 | rs7628806 | 0.366 | 1.000 | 0.394 | 2.09E-06 | 1.12E-04 |
| Nucleus accumbens | DRD3 | rs6762200 | 0.363 | 0.995 | 0.394 | 2.09E-06 | 1.12E-04 |
| Nucleus accumbens | DRD3 | rs2630350 | 0.931 | 1.000 | -0.391 | 2.52E-06 | 1.30E-04 |
| Nucleus accumbens | DRD3 | rs2630351 | 0.931 | 1.000 | -0.391 | 2.52E-06 | 1.30E-04 |
| Nucleus accumbens | DRD3 | rs7625282 | 0.277 | 1.000 | 0.390 | 2.77E-06 | 1.41E-04 |
| Nucleus accumbens | DRD3 | rs9942018 | 0.368 | 0.995 | 0.387 | 3.59E-06 | 1.64E-04 |
| Nucleus accumbens | DRD3 | rs112115712 | 0.057 | 1.000 | 0.377 | 6.05E-06 | 2.12E-04 |
| Nucleus accumbens | DRD3 | rs77489111 | 0.057 | 1.000 | 0.377 | 6.05E-06 | 2.12E-04 |
| Nucleus accumbens | DRD3 | rs111522445 | 0.057 | 1.000 | 0.377 | 6.05E-06 | 2.12E-04 |
| Nucleus accumbens | DRD3 | rs112718054 | 0.057 | 1.000 | 0.377 | 6.05E-06 | 2.12E-04 |
| Nucleus accumbens | DRD3 | rs112529001 | 0.057 | 1.000 | 0.377 | 6.05E-06 | 2.12E-04 |
| Nucleus accumbens | DRD3 | rs76132833 | 0.057 | 1.000 | 0.377 | 6.05E-06 | 2.12E-04 |
| Nucleus accumbens | DRD3 | rs113029186 | 0.057 | 1.000 | 0.377 | 6.05E-06 | 2.12E-04 |
| Nucleus accumbens | DRD3 | rs1486008 | 0.057 | 1.000 | 0.377 | 6.05E-06 | 2.12E-04 |
| Nucleus accumbens | DRD3 | rs1486009 | 0.057 | 1.000 | 0.377 | 6.05E-06 | 2.12E-04 |
| Nucleus accumbens | DRD3 | rs1486010 | 0.057 | 1.000 | 0.377 | 6.05E-06 | 2.12E-04 |
| Nucleus accumbens | DRD3 | rs16822416 | 0.057 | 1.000 | 0.377 | 6.05E-06 | 2.12E-04 |
| Nucleus accumbens | DRD3 | rs3732783 | 0.057 | 1.000 | 0.377 | 6.05E-06 | 2.12E-04 |
| Nucleus accumbens | DRD3 | rs1486011 | 0.057 | 1.000 | 0.377 | 6.05E-06 | 2.12E-04 |
| Nucleus accumbens | DRD3 | rs113325091 | 0.057 | 1.000 | 0.377 | 6.05E-06 | 2.12E-04 |
| Nucleus accumbens | DRD3 | rs74513490 | 0.057 | 1.000 | 0.377 | 6.05E-06 | 2.12E-04 |
| Nucleus accumbens | DRD3 | rs36211798 | 0.057 | 1.000 | 0.377 | 6.05E-06 | 2.12E-04 |
| Nucleus accumbens | DRD3 | rs76459808 | 0.057 | 1.000 | 0.377 | 6.05E-06 | 2.12E-04 |
| Nucleus accumbens | DRD3 | rs34008605 | 0.057 | 1.000 | 0.377 | 6.05E-06 | 2.12E-04 |
| Nucleus accumbens | DRD3 | rs36212177 | 0.057 | 1.000 | 0.377 | 6.05E-06 | 2.12E-04 |
| Nucleus accumbens | DRD3 | rs75811952 | 0.057 | 1.000 | 0.377 | 6.05E-06 | 2.12E-04 |
| Nucleus accumbens | DRD3 | rs74988970 | 0.057 | 1.000 | 0.377 | 6.05E-06 | 2.12E-04 |
| Nucleus accumbens | DRD3 | rs36211796 | 0.055 | 0.995 | 0.376 | 7.13E-06 | 2.32E-04 |
| Nucleus accumbens | DRD3 | rs36212175 | 0.054 | 1.000 | 0.372 | 8.47E-06 | 2.71E-04 |
| Nucleus accumbens | DRD3 | rs2134655 | 0.255 | 1.000 | -0.367 | 1.12E-05 | 3.39E-04 |
| Nucleus accumbens | DRD3 | rs9813633 | 0.059 | 1.000 | 0.361 | 1.55E-05 | 4.51E-04 |
| Nucleus accumbens | DRD3 | rs145438954 | 0.057 | 1.000 | 0.355 | 2.18E-05 | 5.39E-04 |
| Nucleus accumbens | DRD3 | rs36212182 | 0.057 | 1.000 | 0.355 | 2.18E-05 | 5.39E-04 |
| Nucleus accumbens | DRD3 | rs1354348 | 0.057 | 1.000 | 0.355 | 2.18E-05 | 5.39E-04 |
| Nucleus accumbens | DRD3 | rs3773679 | 0.366 | 1.000 | -0.341 | 4.77E-05 | 1.05E-03 |
| Nucleus accumbens | DRD3 | rs9824856 | 0.958 | 1.000 | -0.340 | 5.09E-05 | 1.09E-03 |
| Nucleus accumbens | DRD3 | rs2654754 | 0.958 | 1.000 | -0.340 | 5.09E-05 | 1.09E-03 |
| Nucleus accumbens | DRD3 | rs4592996 | 0.958 | 0.995 | -0.341 | 5.33E-05 | 1.14E-03 |
| Nucleus accumbens | DRD3 | rs6778623 | 0.055 | 0.995 | 0.340 | 5.41E-05 | 1.15E-03 |
| Nucleus accumbens | DRD3 | rs3732790 | 0.376 | 1.000 | -0.333 | 7.49E-05 | 1.45E-03 |
| Nucleus accumbens | DRD3 | rs963468 | 0.371 | 0.995 | -0.334 | 7.67E-05 | 1.48E-03 |
| Nucleus accumbens | DRD3 | rs72946614 | 0.059 | 1.000 | 0.316 | 1.82E-04 | 2.99E-03 |
| Nucleus accumbens | DRD3 | rs73856267 | 0.059 | 1.000 | 0.316 | 1.82E-04 | 2.99E-03 |
| Nucleus accumbens | DRD3 | rs9283560 | 0.059 | 1.000 | 0.316 | 1.82E-04 | 2.99E-03 |
| Nucleus accumbens | DRD3 | rs72944605 | 0.015 | 1.000 | 0.286 | 7.49E-04 | 9.65E-03 |
| Nucleus accumbens | DRD3 | rs72944609 | 0.015 | 1.000 | 0.286 | 7.49E-04 | 9.65E-03 |
| Nucleus accumbens | DRD3 | rs72944611 | 0.015 | 1.000 | 0.286 | 7.49E-04 | 9.65E-03 |
| Nucleus accumbens | DRD3 | rs9825563 | 0.319 | 1.000 | 0.280 | 9.61E-04 | 1.18E-02 |
| Nucleus accumbens | DRD3 | rs7631639 | 0.317 | 1.000 | 0.280 | 9.61E-04 | 1.18E-02 |
| Nucleus accumbens | DRD3 | rs78622784 | 0.059 | 1.000 | -0.280 | 9.67E-04 | 1.18E-02 |
| Nucleus accumbens | DRD3 | rs149642480 | 0.015 | 1.000 | 0.262 | 2.04E-03 | 2.33E-02 |
| Nucleus accumbens | DRD3 | rs143349368 | 0.015 | 1.000 | 0.262 | 2.04E-03 | 2.33E-02 |
| Nucleus accumbens | DRD3 | rs12490922 | 0.966 | 0.950 | -0.270 | 2.08E-03 | 2.35E-02 |
| Nucleus accumbens | DRD3 | rs73235906 | 0.035 | 1.000 | -0.258 | 2.45E-03 | 2.69E-02 |
| Nucleus accumbens | DRD3 | rs9878569 | 0.032 | 1.000 | 0.257 | 2.56E-03 | 2.75E-02 |
| Nucleus accumbens | DRD3 | rs553799663 | 0.035 | 1.000 | 0.257 | 2.56E-03 | 2.75E-02 |
| Nucleus accumbens | DRD3 | rs9288991 | 0.035 | 1.000 | 0.257 | 2.56E-03 | 2.75E-02 |
| Nucleus accumbens | DRD3 | rs9837524 | 0.035 | 1.000 | 0.257 | 2.56E-03 | 2.75E-02 |
| Nucleus accumbens | DRD3 | rs9859762 | 0.035 | 1.000 | 0.257 | 2.56E-03 | 2.75E-02 |
| Nucleus accumbens | DRD3 | rs9828046 | 0.032 | 1.000 | 0.257 | 2.56E-03 | 2.75E-02 |
| Nucleus accumbens | DRD3 | rs9288993 | 0.035 | 1.000 | 0.257 | 2.56E-03 | 2.75E-02 |
| Nucleus accumbens | DRD3 | rs28419745 | 0.035 | 1.000 | 0.257 | 2.56E-03 | 2.75E-02 |
| Nucleus accumbens | DRD3 | rs6786450 | 0.035 | 1.000 | 0.257 | 2.56E-03 | 2.75E-02 |
| Nucleus accumbens | DRD3 | rs11929108 | 0.035 | 1.000 | 0.257 | 2.56E-03 | 2.75E-02 |
| Nucleus accumbens | DRD3 | rs11921500 | 0.035 | 1.000 | 0.257 | 2.56E-03 | 2.75E-02 |
| Nucleus accumbens | DRD3 | rs7629948 | 0.035 | 1.000 | 0.257 | 2.56E-03 | 2.75E-02 |
| Nucleus accumbens | DRD3 | rs9881381 | 0.032 | 0.995 | 0.257 | 2.63E-03 | 2.79E-02 |
| Nucleus accumbens | DRD3 | rs9855387 | 0.035 | 0.995 | 0.256 | 2.74E-03 | 2.87E-02 |
| Nucleus accumbens | DRD3 | rs324041 | 0.988 | 1.000 | -0.253 | 2.99E-03 | 3.12E-02 |
| Nucleus accumbens | DRD3 | rs111704809 | 0.039 | 0.946 | 0.262 | 3.01E-03 | 3.13E-02 |
| Nucleus accumbens | DRD3 | rs561209701 | 0.034 | 0.950 | 0.262 | 3.07E-03 | 3.19E-02 |
| Nucleus accumbens | DRD3 | rs113574410 | 0.038 | 0.965 | 0.259 | 3.09E-03 | 3.21E-02 |
| Nucleus accumbens | DRD3 | rs10934253 | 0.960 | 1.000 | -0.249 | 3.50E-03 | 3.57E-02 |
| Nucleus accumbens | DRD3 | rs9682405 | 0.960 | 1.000 | -0.249 | 3.50E-03 | 3.57E-02 |
| Nucleus accumbens | DRD3 | rs9681085 | 0.960 | 1.000 | -0.249 | 3.50E-03 | 3.57E-02 |
| Nucleus accumbens | DRD3 | rs6808291 | 0.960 | 1.000 | -0.249 | 3.50E-03 | 3.57E-02 |
| Nucleus accumbens | DRD3 | rs549293440 | 0.034 | 0.946 | 0.254 | 3.94E-03 | 3.94E-02 |
| Nucleus accumbens | DRD3 | rs571584371 | 0.034 | 0.946 | 0.252 | 4.55E-03 | 4.40E-02 |
| Nucleus accumbens | DRD3 | rs115852660 | 0.054 | 1.000 | -0.240 | 4.90E-03 | 4.57E-02 |
| Nucleus accumbens | DRD3 | rs111466137 | 0.040 | 1.000 | 0.239 | 5.02E-03 | 4.63E-02 |
| Nucleus accumbens | DRD3 | rs72946624 | 0.065 | 0.995 | 0.238 | 5.38E-03 | 4.82E-02 |
| Nucleus accumbens | DRD3 | rs75479423 | 0.035 | 1.000 | 0.232 | 6.61E-03 | 5.36E-02 |
| Nucleus accumbens | DRD3 | rs9880168 | 0.092 | 1.000 | 0.229 | 7.24E-03 | 5.64E-02 |
| Nucleus accumbens | DRD3 | rs57309847 | 0.034 | 0.960 | 0.233 | 7.50E-03 | 5.78E-02 |
| Nucleus accumbens | DRD3 | rs62267126 | 0.032 | 0.931 | 0.234 | 9.60E-03 | 7.03E-02 |
| Nucleus accumbens | DRD3 | rs713152 | 0.027 | 1.000 | 0.220 | 1.01E-02 | 7.28E-02 |
| Nucleus accumbens | DRD3 | rs75970639 | 0.057 | 1.000 | 0.215 | 1.20E-02 | 8.09E-02 |
| Nucleus accumbens | DRD3 | rs79431774 | 0.057 | 1.000 | 0.215 | 1.20E-02 | 8.09E-02 |
| Nucleus accumbens | DRD3 | rs36212514 | 0.027 | 1.000 | 0.215 | 1.21E-02 | 8.09E-02 |
| Nucleus accumbens | DRD3 | rs60585023 | 0.027 | 1.000 | 0.215 | 1.21E-02 | 8.09E-02 |
| Nucleus accumbens | DRD3 | rs17668524 | 0.027 | 1.000 | 0.215 | 1.21E-02 | 8.09E-02 |
| Nucleus accumbens | DRD3 | rs58192132 | 0.027 | 1.000 | 0.215 | 1.21E-02 | 8.09E-02 |
| Nucleus accumbens | DRD3 | rs77068130 | 0.027 | 1.000 | 0.215 | 1.21E-02 | 8.09E-02 |
| Nucleus accumbens | DRD3 | rs17605534 | 0.027 | 1.000 | 0.215 | 1.21E-02 | 8.09E-02 |
| Nucleus accumbens | DRD3 | rs113508308 | 0.027 | 1.000 | 0.215 | 1.21E-02 | 8.09E-02 |
| Nucleus accumbens | DRD3 | rs113705676 | 0.027 | 1.000 | 0.215 | 1.21E-02 | 8.09E-02 |
| Nucleus accumbens | DRD3 | rs17668694 | 0.027 | 1.000 | 0.215 | 1.21E-02 | 8.09E-02 |
| Nucleus accumbens | DRD3 | rs12495190 | 0.027 | 1.000 | 0.215 | 1.21E-02 | 8.09E-02 |
| Nucleus accumbens | DRD3 | rs12491239 | 0.027 | 1.000 | 0.215 | 1.21E-02 | 8.09E-02 |
| Nucleus accumbens | DRD3 | rs112347350 | 0.027 | 1.000 | 0.215 | 1.21E-02 | 8.09E-02 |
| Nucleus accumbens | DRD3 | rs71653627 | 0.032 | 1.000 | 0.213 | 1.27E-02 | 8.30E-02 |
| Nucleus accumbens | DRD3 | rs75859127 | 0.032 | 1.000 | 0.213 | 1.27E-02 | 8.30E-02 |
| Nucleus accumbens | DRD3 | rs78790093 | 0.032 | 1.000 | 0.213 | 1.27E-02 | 8.30E-02 |
| Nucleus accumbens | DRD3 | rs36212516 | 0.032 | 1.000 | 0.213 | 1.27E-02 | 8.30E-02 |
| Nucleus accumbens | DRD3 | rs111976689 | 0.031 | 0.946 | 0.222 | 1.30E-02 | 8.48E-02 |
| Nucleus accumbens | DRD3 | rs77506468 | 0.012 | 1.000 | 0.211 | 1.35E-02 | 8.69E-02 |
| Nucleus accumbens | DRD3 | rs76545251 | 0.012 | 1.000 | 0.211 | 1.35E-02 | 8.69E-02 |
| Nucleus accumbens | DRD3 | rs115313126 | 0.012 | 1.000 | 0.211 | 1.35E-02 | 8.69E-02 |
| Nucleus accumbens | DRD3 | rs12633630 | 0.214 | 0.995 | 0.211 | 1.39E-02 | 8.88E-02 |
| Nucleus accumbens | DRD3 | rs76597298 | 0.025 | 1.000 | 0.210 | 1.43E-02 | 9.11E-02 |
| Nucleus accumbens | DRD3 | rs115195253 | 0.025 | 1.000 | 0.210 | 1.43E-02 | 9.11E-02 |
| Nucleus accumbens | DRD3 | rs147240145 | 0.030 | 1.000 | -0.206 | 1.61E-02 | 9.94E-02 |
| Nucleus accumbens | DRD3 | rs77348312 | 0.027 | 1.000 | 0.205 | 1.64E-02 | 1.01E-01 |
| Nucleus accumbens | DRD3 | rs76465829 | 0.045 | 1.000 | -0.201 | 1.92E-02 | 1.11E-01 |
| Nucleus accumbens | DRD3 | rs1800828 | 0.238 | 1.000 | 0.199 | 2.03E-02 | 1.14E-01 |
| Nucleus accumbens | DRD3 | rs7633291 | 0.220 | 1.000 | 0.198 | 2.10E-02 | 1.17E-01 |
| Nucleus accumbens | DRD3 | rs60730451 | 0.220 | 1.000 | 0.198 | 2.10E-02 | 1.17E-01 |
| Nucleus accumbens | DRD3 | rs12330143 | 0.027 | 1.000 | 0.197 | 2.18E-02 | 1.20E-01 |
| Nucleus accumbens | DRD3 | rs9288992 | 0.027 | 1.000 | 0.197 | 2.18E-02 | 1.20E-01 |
| Nucleus accumbens | DRD3 | rs76227642 | 0.037 | 1.000 | 0.196 | 2.22E-02 | 1.22E-01 |
| Nucleus accumbens | DRD3 | rs61380137 | 0.037 | 0.995 | 0.196 | 2.26E-02 | 1.23E-01 |
| Nucleus accumbens | DRD3 | rs73232565 | 0.171 | 1.000 | 0.182 | 3.44E-02 | 1.68E-01 |
| Nucleus accumbens | DRD3 | rs11715906 | 0.552 | 1.000 | 0.181 | 3.45E-02 | 1.69E-01 |
| Nucleus accumbens | DRD3 | rs1486012 | 0.552 | 1.000 | 0.181 | 3.45E-02 | 1.69E-01 |
| Nucleus accumbens | DRD3 | rs12636133 | 0.552 | 1.000 | 0.181 | 3.45E-02 | 1.69E-01 |
| Nucleus accumbens | DRD3 | rs9871472 | 0.030 | 1.000 | 0.178 | 3.87E-02 | 1.84E-01 |
| Nucleus accumbens | DRD3 | rs9811326 | 0.030 | 1.000 | 0.178 | 3.87E-02 | 1.84E-01 |
| Nucleus accumbens | DRD3 | rs61595610 | 0.030 | 1.000 | 0.178 | 3.87E-02 | 1.84E-01 |
| Nucleus accumbens | DRD3 | rs6806217 | 0.030 | 1.000 | 0.178 | 3.87E-02 | 1.84E-01 |
| Nucleus accumbens | DRD3 | rs1800827 | 0.030 | 1.000 | 0.178 | 3.87E-02 | 1.84E-01 |
| Nucleus accumbens | DRD3 | rs72944698 | 0.030 | 1.000 | 0.178 | 3.87E-02 | 1.84E-01 |
| Nucleus accumbens | DRD3 | rs6787134 | 0.116 | 1.000 | 0.174 | 4.28E-02 | 1.94E-01 |
| Nucleus accumbens | DRD5 | rs6842850 | 0.767 | 1.000 | -0.215 | 2.81E-02 | 1.45E-01 |
| Nucleus accumbens | DRD5 | rs140290358 | 0.196 | 1.000 | 0.202 | 4.03E-02 | 1.88E-01 |
| Nucleus accumbens | SLC6A3 | rs2455391 | 0.248 | 1.000 | 0.357 | 2.18E-02 | 1.20E-01 |
| Nucleus accumbens | SLC6A3 | rs2550956 | 0.255 | 1.000 | 0.357 | 2.18E-02 | 1.20E-01 |
| Nucleus accumbens | SLC6A3 | rs2550950 | 0.255 | 1.000 | 0.357 | 2.18E-02 | 1.20E-01 |
| Nucleus accumbens | SLC6A3 | rs11564769 | 0.092 | 1.000 | 0.333 | 3.34E-02 | 1.64E-01 |
| Nucleus accumbens | SLC6A3 | rs56982424 | 0.012 | 1.000 | 0.330 | 3.49E-02 | 1.70E-01 |
| Nucleus accumbens | SLC6A3 | rs10074171 | 0.297 | 0.965 | 0.337 | 3.61E-02 | 1.75E-01 |
| Nucleus accumbens | SLC6A3 | rs40184 | 0.411 | 1.000 | 0.326 | 3.72E-02 | 1.79E-01 |
| Prefrontal cortex | ANKK1 | rs4938012 | 0.677 | 1.000 | -0.393 | 4.80E-06 | 1.41E-04 |
| Prefrontal cortex | ANKK1 | rs7945132 | 0.677 | 1.000 | -0.393 | 4.80E-06 | 1.41E-04 |
| Prefrontal cortex | ANKK1 | rs4938013 | 0.677 | 1.000 | -0.393 | 4.80E-06 | 1.41E-04 |
| Prefrontal cortex | ANKK1 | rs7123797 | 0.644 | 0.994 | -0.379 | 1.22E-05 | 3.18E-04 |
| Prefrontal cortex | ANKK1 | rs17115439 | 0.640 | 1.000 | -0.377 | 1.28E-05 | 3.29E-04 |
| Prefrontal cortex | ANKK1 | rs4938015 | 0.644 | 0.994 | -0.377 | 1.28E-05 | 3.29E-04 |
| Prefrontal cortex | ANKK1 | rs10891546 | 0.640 | 1.000 | -0.377 | 1.28E-05 | 3.29E-04 |
| Prefrontal cortex | ANKK1 | rs877138 | 0.677 | 1.000 | -0.353 | 4.60E-05 | 8.12E-04 |
| Prefrontal cortex | ANKK1 | rs3897584 | 0.678 | 0.994 | -0.354 | 4.87E-05 | 8.57E-04 |
| Prefrontal cortex | ANKK1 | rs4601821 | 0.671 | 1.000 | -0.336 | 1.15E-04 | 1.85E-03 |
| Prefrontal cortex | ANKK1 | rs10891543 | 0.671 | 1.000 | -0.336 | 1.15E-04 | 1.85E-03 |
| Prefrontal cortex | ANKK1 | rs2734847 | 0.794 | 1.000 | -0.330 | 1.55E-04 | 2.40E-03 |
| Prefrontal cortex | ANKK1 | rs2234689 | 0.794 | 1.000 | -0.330 | 1.55E-04 | 2.40E-03 |
| Prefrontal cortex | ANKK1 | rs2734848 | 0.763 | 1.000 | -0.316 | 2.95E-04 | 4.25E-03 |
| Prefrontal cortex | ANKK1 | rs12422191 | 0.094 | 1.000 | 0.258 | 3.43E-03 | 2.77E-02 |
| Prefrontal cortex | ANKK1 | rs61492892 | 0.158 | 0.994 | -0.242 | 6.05E-03 | 4.37E-02 |
| Prefrontal cortex | ANKK1 | rs12224538 | 0.163 | 1.000 | -0.242 | 6.05E-03 | 4.37E-02 |
| Prefrontal cortex | ANKK1 | rs4590907 | 0.163 | 1.000 | -0.242 | 6.05E-03 | 4.37E-02 |
| Prefrontal cortex | ANKK1 | rs4938014 | 0.163 | 1.000 | -0.242 | 6.05E-03 | 4.37E-02 |
| Prefrontal cortex | ANKK1 | rs10891542 | 0.160 | 1.000 | -0.226 | 1.08E-02 | 6.49E-02 |
| Prefrontal cortex | ANKK1 | rs10891541 | 0.161 | 0.994 | -0.225 | 1.13E-02 | 6.72E-02 |
| Prefrontal cortex | ANKK1 | rs10891545 | 0.197 | 1.000 | -0.202 | 2.30E-02 | 1.18E-01 |
| Prefrontal cortex | ANKK1 | rs138823496 | 0.014 | 1.000 | -0.197 | 2.67E-02 | 1.33E-01 |
| Prefrontal cortex | ANKK1 | rs7105965 | 0.020 | 0.994 | 0.197 | 2.68E-02 | 1.33E-01 |
| Prefrontal cortex | ANKK1 | rs75698063 | 0.063 | 1.000 | -0.178 | 4.50E-02 | 1.93E-01 |
| Prefrontal cortex | ANKK1 | rs75249539 | 0.063 | 1.000 | -0.178 | 4.50E-02 | 1.93E-01 |
| Prefrontal cortex | ANKK1 | rs73557236 | 0.094 | 1.000 | -0.177 | 4.70E-02 | 1.99E-01 |
| Prefrontal cortex | DBH | rs12343735 | 0.031 | 1.000 | 0.295 | 1.45E-04 | 2.27E-03 |
| Prefrontal cortex | DBH | rs9657707 | 0.037 | 1.000 | 0.295 | 1.48E-04 | 2.32E-03 |
| Prefrontal cortex | DBH | rs3025402 | 0.029 | 1.000 | 0.282 | 2.83E-04 | 4.20E-03 |
| Prefrontal cortex | DBH | rs129922 | 0.751 | 1.000 | 0.277 | 3.80E-04 | 5.06E-03 |
| Prefrontal cortex | DBH | rs7035577 | 0.034 | 1.000 | 0.269 | 5.67E-04 | 6.84E-03 |
| Prefrontal cortex | DBH | rs3025419 | 0.023 | 1.000 | 0.265 | 6.67E-04 | 7.94E-03 |
| Prefrontal cortex | DBH | rs73559959 | 0.023 | 1.000 | 0.265 | 6.67E-04 | 7.94E-03 |
| Prefrontal cortex | DBH | rs129883 | 0.760 | 1.000 | 0.263 | 7.42E-04 | 8.77E-03 |
| Prefrontal cortex | DBH | rs73662170 | 0.031 | 1.000 | 0.262 | 7.77E-04 | 9.02E-03 |
| Prefrontal cortex | DBH | rs7862391 | 0.031 | 1.000 | 0.262 | 7.77E-04 | 9.02E-03 |
| Prefrontal cortex | DBH | rs77358884 | 0.011 | 1.000 | 0.262 | 7.90E-04 | 9.11E-03 |
| Prefrontal cortex | DBH | rs3025413 | 0.034 | 1.000 | 0.230 | 3.38E-03 | 2.74E-02 |
| Prefrontal cortex | DBH | rs12003344 | 0.034 | 1.000 | 0.230 | 3.38E-03 | 2.74E-02 |
| Prefrontal cortex | DBH | rs7855248 | 0.466 | 1.000 | 0.225 | 4.13E-03 | 3.17E-02 |
| Prefrontal cortex | DBH | rs7876027 | 0.037 | 1.000 | 0.225 | 4.16E-03 | 3.19E-02 |
| Prefrontal cortex | DBH | rs7871224 | 0.469 | 1.000 | 0.220 | 4.96E-03 | 3.67E-02 |
| Prefrontal cortex | DBH | rs10761412 | 0.480 | 1.000 | 0.218 | 5.55E-03 | 4.05E-02 |
| Prefrontal cortex | DBH | rs3025391 | 0.020 | 1.000 | 0.217 | 5.78E-03 | 4.20E-02 |
| Prefrontal cortex | DBH | rs2097629 | 0.449 | 1.000 | 0.213 | 6.61E-03 | 4.65E-02 |
| Prefrontal cortex | DBH | rs129913 | 0.020 | 1.000 | 0.213 | 6.75E-03 | 4.73E-02 |
| Prefrontal cortex | DBH | rs74565558 | 0.023 | 1.000 | 0.212 | 7.00E-03 | 4.76E-02 |
| Prefrontal cortex | DBH | rs7854480 | 0.600 | 1.000 | 0.210 | 7.51E-03 | 4.95E-02 |
| Prefrontal cortex | DBH | rs2797853 | 0.691 | 1.000 | 0.208 | 7.97E-03 | 5.19E-02 |
| Prefrontal cortex | DBH | rs73559912 | 0.020 | 1.000 | 0.207 | 8.59E-03 | 5.48E-02 |
| Prefrontal cortex | DBH | rs10993949 | 0.023 | 1.000 | 0.204 | 9.47E-03 | 5.87E-02 |
| Prefrontal cortex | DBH | rs113498112 | 0.026 | 1.000 | 0.204 | 9.57E-03 | 5.87E-02 |
| Prefrontal cortex | DBH | rs73662171 | 0.026 | 1.000 | 0.204 | 9.57E-03 | 5.87E-02 |
| Prefrontal cortex | DBH | rs10121827 | 0.026 | 1.000 | 0.204 | 9.57E-03 | 5.87E-02 |
| Prefrontal cortex | DBH | rs142682914 | 0.026 | 1.000 | 0.204 | 9.57E-03 | 5.87E-02 |
| Prefrontal cortex | DBH | rs111774197 | 0.026 | 1.000 | 0.204 | 9.57E-03 | 5.87E-02 |
| Prefrontal cortex | DBH | rs115396855 | 0.026 | 1.000 | 0.204 | 9.57E-03 | 5.87E-02 |
| Prefrontal cortex | DBH | rs78776711 | 0.026 | 1.000 | 0.204 | 9.57E-03 | 5.87E-02 |
| Prefrontal cortex | DBH | rs3025395 | 0.023 | 1.000 | 0.203 | 9.81E-03 | 5.96E-02 |
| Prefrontal cortex | DBH | rs17150766 | 0.020 | 1.000 | 0.201 | 1.07E-02 | 6.47E-02 |
| Prefrontal cortex | DBH | rs116929748 | 0.017 | 1.000 | 0.200 | 1.09E-02 | 6.56E-02 |
| Prefrontal cortex | DBH | rs13306308 | 0.017 | 1.000 | 0.200 | 1.09E-02 | 6.56E-02 |
| Prefrontal cortex | DBH | rs117053657 | 0.017 | 1.000 | -0.200 | 1.09E-02 | 6.56E-02 |
| Prefrontal cortex | DBH | rs113366361 | 0.017 | 1.000 | 0.200 | 1.11E-02 | 6.63E-02 |
| Prefrontal cortex | DBH | rs3025375 | 0.017 | 1.000 | 0.200 | 1.11E-02 | 6.63E-02 |
| Prefrontal cortex | DBH | rs2097628 | 0.457 | 1.000 | 0.194 | 1.36E-02 | 7.92E-02 |
| Prefrontal cortex | DBH | rs3025422 | 0.026 | 1.000 | 0.193 | 1.44E-02 | 8.26E-02 |
| Prefrontal cortex | DBH | rs129887 | 0.221 | 0.994 | 0.193 | 1.45E-02 | 8.34E-02 |
| Prefrontal cortex | DBH | rs1108581 | 0.220 | 1.000 | 0.191 | 1.54E-02 | 8.70E-02 |
| Prefrontal cortex | DBH | rs2519153 | 0.306 | 1.000 | -0.187 | 1.75E-02 | 9.43E-02 |
| Prefrontal cortex | DBH | rs1611128 | 0.306 | 1.000 | -0.187 | 1.75E-02 | 9.43E-02 |
| Prefrontal cortex | DBH | rs129882 | 0.203 | 1.000 | 0.186 | 1.81E-02 | 9.73E-02 |
| Prefrontal cortex | DBH | rs147644100 | 0.020 | 1.000 | 0.186 | 1.85E-02 | 9.90E-02 |
| Prefrontal cortex | DBH | rs5322 | 0.020 | 1.000 | 0.186 | 1.85E-02 | 9.90E-02 |
| Prefrontal cortex | DBH | rs73662176 | 0.011 | 1.000 | 0.183 | 2.05E-02 | 1.08E-01 |
| Prefrontal cortex | DBH | rs80350133 | 0.011 | 1.000 | 0.183 | 2.05E-02 | 1.08E-01 |
| Prefrontal cortex | DBH | rs7043939 | 0.011 | 1.000 | 0.183 | 2.05E-02 | 1.08E-01 |
| Prefrontal cortex | DBH | rs129930 | 0.586 | 1.000 | 0.182 | 2.08E-02 | 1.09E-01 |
| Prefrontal cortex | DBH | rs129931 | 0.591 | 1.000 | 0.181 | 2.20E-02 | 1.14E-01 |
| Prefrontal cortex | DBH | rs7851898 | 0.324 | 0.989 | 0.181 | 2.27E-02 | 1.17E-01 |
| Prefrontal cortex | DBH | rs129886 | 0.214 | 1.000 | 0.170 | 3.08E-02 | 1.46E-01 |
| Prefrontal cortex | DBH | rs10993948 | 0.020 | 1.000 | 0.170 | 3.13E-02 | 1.47E-01 |
| Prefrontal cortex | DBH | rs3025425 | 0.020 | 1.000 | 0.170 | 3.13E-02 | 1.47E-01 |
| Prefrontal cortex | DBH | rs129914 | 0.020 | 1.000 | 0.170 | 3.13E-02 | 1.47E-01 |
| Prefrontal cortex | DBH | rs4531 | 0.103 | 1.000 | 0.168 | 3.29E-02 | 1.54E-01 |
| Prefrontal cortex | DBH | rs1611127 | 0.103 | 1.000 | 0.168 | 3.29E-02 | 1.54E-01 |
| Prefrontal cortex | DBH | rs3025383 | 0.189 | 1.000 | 0.165 | 3.64E-02 | 1.65E-01 |
| Prefrontal cortex | DBH | rs7033395 | 0.207 | 0.994 | 0.165 | 3.72E-02 | 1.68E-01 |
| Prefrontal cortex | DBH | rs129884 | 0.184 | 0.994 | 0.165 | 3.75E-02 | 1.69E-01 |
| Prefrontal cortex | DBH | rs2073837 | 0.363 | 1.000 | 0.160 | 4.21E-02 | 1.83E-01 |
| Prefrontal cortex | DBH | rs4979627 | 0.221 | 0.994 | 0.158 | 4.65E-02 | 1.98E-01 |
| Prefrontal cortex | DRD1 | rs28465440 | 0.114 | 1.000 | 0.475 | 2.92E-08 | 1.57E-06 |
| Prefrontal cortex | DRD1 | rs35916350 | 0.086 | 1.000 | 0.471 | 3.91E-08 | 2.06E-06 |
| Prefrontal cortex | DRD1 | rs12518222 | 0.098 | 0.994 | 0.449 | 2.13E-07 | 8.43E-06 |
| Prefrontal cortex | DRD1 | rs5326 | 0.106 | 1.000 | 0.416 | 1.71E-06 | 5.92E-05 |
| Prefrontal cortex | DRD1 | rs265977 | 0.149 | 0.994 | 0.307 | 5.48E-04 | 6.64E-03 |
| Prefrontal cortex | DRD1 | rs265976 | 0.177 | 1.000 | 0.271 | 2.40E-03 | 2.07E-02 |
| Prefrontal cortex | DRD1 | rs10463051 | 0.140 | 1.000 | 0.270 | 2.49E-03 | 2.13E-02 |
| Prefrontal cortex | DRD1 | rs703748 | 0.349 | 1.000 | -0.266 | 2.94E-03 | 2.47E-02 |
| Prefrontal cortex | DRD1 | rs185482023 | 0.017 | 1.000 | -0.251 | 5.19E-03 | 3.82E-02 |
| Prefrontal cortex | DRD1 | rs4867798 | 0.264 | 0.994 | 0.243 | 6.89E-03 | 4.73E-02 |
| Prefrontal cortex | DRD1 | rs62388319 | 0.029 | 1.000 | -0.235 | 8.80E-03 | 5.55E-02 |
| Prefrontal cortex | DRD1 | rs56000379 | 0.029 | 1.000 | -0.235 | 8.80E-03 | 5.55E-02 |
| Prefrontal cortex | DRD1 | rs62388322 | 0.020 | 1.000 | -0.231 | 1.01E-02 | 6.11E-02 |
| Prefrontal cortex | DRD1 | rs251937 | 0.370 | 0.989 | -0.224 | 1.36E-02 | 7.92E-02 |
| Prefrontal cortex | DRD1 | rs4867796 | 0.174 | 1.000 | -0.207 | 2.15E-02 | 1.12E-01 |
| Prefrontal cortex | DRD1 | rs835540 | 0.310 | 0.994 | -0.205 | 2.37E-02 | 1.21E-01 |
| Prefrontal cortex | DRD2 | rs2471855 | 0.129 | 1.000 | 0.305 | 5.83E-04 | 7.02E-03 |
| Prefrontal cortex | DRD2 | rs2471857 | 0.131 | 1.000 | 0.301 | 6.93E-04 | 8.23E-03 |
| Prefrontal cortex | DRD2 | rs55900980 | 0.126 | 1.000 | 0.298 | 7.61E-04 | 8.91E-03 |
| Prefrontal cortex | DRD2 | rs2283265 | 0.126 | 1.000 | 0.298 | 7.61E-04 | 8.91E-03 |
| Prefrontal cortex | DRD2 | rs2075654 | 0.126 | 1.000 | 0.298 | 7.61E-04 | 8.91E-03 |
| Prefrontal cortex | DRD2 | rs1079727 | 0.126 | 1.000 | 0.298 | 7.61E-04 | 8.91E-03 |
| Prefrontal cortex | DRD2 | rs2734836 | 0.126 | 1.000 | 0.298 | 7.61E-04 | 8.91E-03 |
| Prefrontal cortex | DRD2 | rs1962262 | 0.126 | 1.000 | 0.298 | 7.61E-04 | 8.91E-03 |
| Prefrontal cortex | DRD2 | rs1079598 | 0.126 | 1.000 | 0.298 | 7.61E-04 | 8.91E-03 |
| Prefrontal cortex | DRD2 | rs1125393 | 0.126 | 0.994 | 0.292 | 1.04E-03 | 1.13E-02 |
| Prefrontal cortex | DRD2 | rs1079597 | 0.134 | 1.000 | 0.290 | 1.08E-03 | 1.14E-02 |
| Prefrontal cortex | DRD2 | rs2471854 | 0.129 | 0.994 | 0.288 | 1.22E-03 | 1.25E-02 |
| Prefrontal cortex | DRD2 | rs1079596 | 0.137 | 1.000 | 0.286 | 1.26E-03 | 1.28E-02 |
| Prefrontal cortex | DRD2 | rs1125394 | 0.137 | 1.000 | 0.286 | 1.26E-03 | 1.28E-02 |
| Prefrontal cortex | DRD2 | rs11214601 | 0.137 | 1.000 | 0.281 | 1.55E-03 | 1.50E-02 |
| Prefrontal cortex | DRD2 | rs2242593 | 0.137 | 1.000 | 0.281 | 1.55E-03 | 1.50E-02 |
| Prefrontal cortex | DRD2 | rs2471851 | 0.126 | 1.000 | 0.279 | 1.71E-03 | 1.63E-02 |
| Prefrontal cortex | DRD2 | rs7131627 | 0.575 | 0.994 | -0.268 | 2.77E-03 | 2.35E-02 |
| Prefrontal cortex | DRD2 | rs7131465 | 0.575 | 0.994 | -0.268 | 2.77E-03 | 2.35E-02 |
| Prefrontal cortex | DRD2 | rs1076560 | 0.131 | 1.000 | 0.263 | 3.20E-03 | 2.63E-02 |
| Prefrontal cortex | DRD2 | rs11214599 | 0.134 | 1.000 | 0.261 | 3.46E-03 | 2.77E-02 |
| Prefrontal cortex | DRD2 | rs2511520 | 0.134 | 1.000 | 0.261 | 3.46E-03 | 2.77E-02 |
| Prefrontal cortex | DRD2 | rs2242591 | 0.134 | 1.000 | 0.261 | 3.46E-03 | 2.77E-02 |
| Prefrontal cortex | DRD2 | rs6278 | 0.134 | 1.000 | 0.261 | 3.46E-03 | 2.77E-02 |
| Prefrontal cortex | DRD2 | rs1124491 | 0.134 | 1.000 | 0.261 | 3.46E-03 | 2.77E-02 |
| Prefrontal cortex | DRD2 | rs1079595 | 0.134 | 1.000 | 0.261 | 3.46E-03 | 2.77E-02 |
| Prefrontal cortex | DRD2 | rs1079594 | 0.134 | 1.000 | 0.261 | 3.46E-03 | 2.77E-02 |
| Prefrontal cortex | DRD2 | rs7103679 | 0.869 | 1.000 | -0.252 | 4.78E-03 | 3.56E-02 |
| Prefrontal cortex | DRD2 | rs7131440 | 0.571 | 1.000 | -0.250 | 5.09E-03 | 3.76E-02 |
| Prefrontal cortex | DRD2 | rs7131681 | 0.571 | 1.000 | -0.250 | 5.09E-03 | 3.76E-02 |
| Prefrontal cortex | DRD2 | rs7350522 | 0.126 | 1.000 | 0.243 | 6.52E-03 | 4.60E-02 |
| Prefrontal cortex | DRD2 | rs2734839 | 0.591 | 1.000 | -0.240 | 7.37E-03 | 4.88E-02 |
| Prefrontal cortex | DRD2 | rs2734837 | 0.591 | 1.000 | -0.240 | 7.37E-03 | 4.88E-02 |
| Prefrontal cortex | DRD2 | rs11214607 | 0.123 | 1.000 | 0.234 | 8.80E-03 | 5.55E-02 |
| Prefrontal cortex | DRD2 | rs4648319 | 0.123 | 1.000 | 0.234 | 8.80E-03 | 5.55E-02 |
| Prefrontal cortex | DRD2 | rs75644434 | 0.031 | 1.000 | 0.233 | 9.09E-03 | 5.68E-02 |
| Prefrontal cortex | DRD2 | rs117317480 | 0.031 | 1.000 | 0.233 | 9.09E-03 | 5.68E-02 |
| Prefrontal cortex | DRD2 | rs1107162 | 0.578 | 0.994 | -0.219 | 1.49E-02 | 8.52E-02 |
| Prefrontal cortex | DRD2 | rs2234690 | 0.578 | 0.994 | -0.219 | 1.49E-02 | 8.52E-02 |
| Prefrontal cortex | DRD2 | rs11608185 | 0.578 | 0.994 | -0.219 | 1.49E-02 | 8.52E-02 |
| Prefrontal cortex | DRD2 | rs12798900 | 0.566 | 1.000 | -0.217 | 1.57E-02 | 8.81E-02 |
| Prefrontal cortex | DRD2 | rs11214605 | 0.566 | 1.000 | -0.217 | 1.57E-02 | 8.81E-02 |
| Prefrontal cortex | DRD2 | rs12800853 | 0.566 | 1.000 | -0.217 | 1.57E-02 | 8.81E-02 |
| Prefrontal cortex | DRD2 | rs2734832 | 0.572 | 0.994 | -0.216 | 1.62E-02 | 8.87E-02 |
| Prefrontal cortex | DRD2 | rs2734831 | 0.572 | 0.994 | -0.216 | 1.62E-02 | 8.87E-02 |
| Prefrontal cortex | DRD2 | rs80310688 | 0.029 | 1.000 | 0.215 | 1.64E-02 | 8.91E-02 |
| Prefrontal cortex | DRD2 | rs75765286 | 0.029 | 1.000 | 0.215 | 1.64E-02 | 8.91E-02 |
| Prefrontal cortex | DRD2 | rs12364051 | 0.571 | 1.000 | -0.211 | 1.89E-02 | 1.01E-01 |
| Prefrontal cortex | DRD2 | rs2734833 | 0.583 | 1.000 | -0.208 | 2.03E-02 | 1.08E-01 |
| Prefrontal cortex | DRD2 | rs2734838 | 0.574 | 1.000 | -0.202 | 2.47E-02 | 1.24E-01 |
| Prefrontal cortex | DRD2 | rs1800498 | 0.574 | 1.000 | -0.202 | 2.47E-02 | 1.24E-01 |
| Prefrontal cortex | DRD2 | rs2587548 | 0.574 | 1.000 | -0.202 | 2.47E-02 | 1.24E-01 |
| Prefrontal cortex | DRD2 | rs12808482 | 0.574 | 1.000 | -0.202 | 2.47E-02 | 1.24E-01 |
| Prefrontal cortex | DRD2 | rs1076563 | 0.574 | 1.000 | -0.202 | 2.47E-02 | 1.24E-01 |
| Prefrontal cortex | DRD2 | rs1116313 | 0.574 | 1.000 | -0.202 | 2.47E-02 | 1.24E-01 |
| Prefrontal cortex | DRD2 | rs4938017 | 0.403 | 1.000 | 0.201 | 2.49E-02 | 1.25E-01 |
| Prefrontal cortex | DRD2 | rs1800497 | 0.206 | 1.000 | 0.201 | 2.49E-02 | 1.25E-01 |
| Prefrontal cortex | DRD2 | rs12363125 | 0.569 | 1.000 | -0.200 | 2.61E-02 | 1.30E-01 |
| Prefrontal cortex | DRD2 | rs2734835 | 0.569 | 1.000 | -0.200 | 2.61E-02 | 1.30E-01 |
| Prefrontal cortex | DRD2 | rs34554234 | 0.569 | 1.000 | -0.200 | 2.61E-02 | 1.30E-01 |
| Prefrontal cortex | DRD2 | rs11608109 | 0.569 | 1.000 | -0.200 | 2.61E-02 | 1.30E-01 |
| Prefrontal cortex | DRD2 | rs7122246 | 0.569 | 1.000 | -0.200 | 2.61E-02 | 1.30E-01 |
| Prefrontal cortex | DRD2 | rs4587762 | 0.569 | 1.000 | -0.200 | 2.61E-02 | 1.30E-01 |
| Prefrontal cortex | DRD2 | rs11214609 | 0.574 | 1.000 | -0.196 | 2.89E-02 | 1.38E-01 |
| Prefrontal cortex | DRD2 | rs117704709 | 0.017 | 0.994 | 0.193 | 3.22E-02 | 1.51E-01 |
| Prefrontal cortex | DRD2 | rs6277 | 0.497 | 1.000 | -0.190 | 3.49E-02 | 1.60E-01 |
| Prefrontal cortex | DRD2 | rs11214608 | 0.571 | 1.000 | -0.186 | 3.85E-02 | 1.72E-01 |
| Prefrontal cortex | DRD2 | rs10891549 | 0.500 | 1.000 | -0.186 | 3.90E-02 | 1.73E-01 |
| Prefrontal cortex | DRD2 | rs35921774 | 0.023 | 0.994 | -0.181 | 4.43E-02 | 1.90E-01 |
| Prefrontal cortex | DRD3 | rs7629232 | 0.354 | 1.000 | -0.499 | 2.14E-02 | 1.12E-01 |
| Prefrontal cortex | DRD3 | rs9880494 | 0.351 | 0.994 | -0.499 | 2.14E-02 | 1.12E-01 |
| Prefrontal cortex | DRD3 | rs6801068 | 0.351 | 1.000 | -0.499 | 2.14E-02 | 1.12E-01 |
| Prefrontal cortex | DRD3 | rs7628806 | 0.357 | 1.000 | -0.499 | 2.14E-02 | 1.12E-01 |
| Prefrontal cortex | DRD3 | rs6762200 | 0.357 | 1.000 | -0.499 | 2.14E-02 | 1.12E-01 |
| Prefrontal cortex | DRD3 | rs9942018 | 0.357 | 1.000 | -0.499 | 2.14E-02 | 1.12E-01 |
| Prefrontal cortex | DRD3 | rs6778623 | 0.057 | 0.994 | 0.450 | 4.66E-02 | 1.98E-01 |
| Prefrontal cortex | DRD5 | rs6283 | 0.634 | 1.000 | 0.288 | 3.20E-03 | 2.63E-02 |
| Prefrontal cortex | SLC6A3 | rs144980910 | 0.011 | 1.000 | 0.486 | 1.37E-02 | 7.93E-02 |
| Prefrontal cortex | SLC6A3 | rs368124584 | 0.011 | 1.000 | 0.486 | 1.37E-02 | 7.93E-02 |
| Prefrontal cortex | SLC6A3 | rs28363149 | 0.100 | 1.000 | 0.471 | 1.76E-02 | 9.47E-02 |
| Prefrontal cortex | SLC6A3 | rs6869645 | 0.063 | 1.000 | 0.440 | 2.77E-02 | 1.35E-01 |
| Prefrontal cortex | SLC6A3 | rs11564766 | 0.057 | 1.000 | 0.440 | 2.77E-02 | 1.35E-01 |
| Prefrontal cortex | SLC6A3 | rs28363119 | 0.071 | 1.000 | 0.440 | 2.77E-02 | 1.35E-01 |
| Prefrontal cortex | SLC6A3 | rs11133768 | 0.071 | 1.000 | 0.440 | 2.77E-02 | 1.35E-01 |
| Prefrontal cortex | SLC6A3 | rs11564764 | 0.060 | 1.000 | 0.440 | 2.77E-02 | 1.35E-01 |
| Prefrontal cortex | SLC6A3 | rs6876225 | 0.063 | 1.000 | 0.440 | 2.77E-02 | 1.35E-01 |
| Prefrontal cortex | SLC6A3 | rs11564767 | 0.097 | 1.000 | 0.437 | 2.89E-02 | 1.38E-01 |
| Prefrontal cortex | SLC6A3 | rs40184 | 0.403 | 1.000 | 0.427 | 3.33E-02 | 1.55E-01 |
| Prefrontal cortex | SLC6A3 | rs115804091 | 0.026 | 1.000 | 0.403 | 4.56E-02 | 1.95E-01 |
| Prefrontal cortex | SLC6A3 | rs148590368 | 0.026 | 1.000 | 0.403 | 4.56E-02 | 1.95E-01 |
| Substantia nigra | ANKK1 | rs12422191 | 0.075 | 1.000 | 0.316 | 4.35E-03 | 1.10E-01 |
| Substantia nigra | DBH | rs2097628 | 0.404 | 1.000 | 0.328 | 1.48E-03 | 6.23E-02 |
| Substantia nigra | DBH | rs10761412 | 0.421 | 1.000 | 0.328 | 1.50E-03 | 6.23E-02 |
| Substantia nigra | DBH | rs2073836 | 0.373 | 1.000 | 0.327 | 1.56E-03 | 6.23E-02 |
| Substantia nigra | DBH | rs2097629 | 0.399 | 1.000 | 0.323 | 1.80E-03 | 6.23E-02 |
| Substantia nigra | DBH | rs2073833 | 0.474 | 1.000 | 0.322 | 1.86E-03 | 6.24E-02 |
| Substantia nigra | DBH | rs129922 | 0.759 | 1.000 | 0.304 | 3.41E-03 | 9.25E-02 |
| Substantia nigra | DBH | rs129883 | 0.763 | 1.000 | 0.303 | 3.48E-03 | 9.25E-02 |
| Substantia nigra | DBH | rs732833 | 0.465 | 1.000 | 0.302 | 3.62E-03 | 9.47E-02 |
| Substantia nigra | DBH | rs7871224 | 0.412 | 1.000 | 0.295 | 4.56E-03 | 1.13E-01 |
| Substantia nigra | DBH | rs7855248 | 0.412 | 1.000 | 0.295 | 4.56E-03 | 1.13E-01 |
| Substantia nigra | DBH | rs129927 | 0.360 | 1.000 | 0.292 | 4.92E-03 | 1.19E-01 |
| Substantia nigra | DBH | rs2073837 | 0.355 | 1.000 | 0.287 | 5.79E-03 | 1.32E-01 |
| Substantia nigra | DBH | rs2073835 | 0.346 | 1.000 | 0.279 | 7.50E-03 | 1.58E-01 |
| Substantia nigra | DBH | rs129915 | 0.346 | 1.000 | 0.267 | 1.06E-02 | 2.00E-01 |
| Substantia nigra | DRD2 | rs1554929 | 0.500 | 1.000 | -0.356 | 7.25E-04 | 3.82E-02 |
| Substantia nigra | DRD2 | rs11214603 | 0.504 | 1.000 | -0.338 | 1.35E-03 | 6.23E-02 |
| Substantia nigra | DRD2 | rs10891549 | 0.513 | 1.000 | -0.334 | 1.58E-03 | 6.23E-02 |
| Substantia nigra | DRD2 | rs6277 | 0.509 | 1.000 | -0.334 | 1.58E-03 | 6.23E-02 |
| Substantia nigra | DRD2 | rs1125394 | 0.124 | 0.991 | 0.325 | 2.26E-03 | 7.19E-02 |
| Substantia nigra | DRD2 | rs2471857 | 0.123 | 1.000 | 0.317 | 2.80E-03 | 8.47E-02 |
| Substantia nigra | DRD2 | rs2471854 | 0.123 | 1.000 | 0.317 | 2.80E-03 | 8.47E-02 |
| Substantia nigra | DRD2 | rs1079596 | 0.127 | 1.000 | 0.306 | 3.94E-03 | 9.97E-02 |
| Substantia nigra | DRD2 | rs7103679 | 0.886 | 1.000 | -0.295 | 5.55E-03 | 1.31E-01 |
| Substantia nigra | DRD2 | rs117816894 | 0.022 | 1.000 | 0.291 | 6.21E-03 | 1.39E-01 |
| Substantia nigra | DRD2 | rs11214599 | 0.118 | 1.000 | 0.290 | 6.44E-03 | 1.39E-01 |
| Substantia nigra | DRD2 | rs11214601 | 0.118 | 1.000 | 0.290 | 6.44E-03 | 1.39E-01 |
| Substantia nigra | DRD2 | rs2511520 | 0.118 | 1.000 | 0.290 | 6.44E-03 | 1.39E-01 |
| Substantia nigra | DRD2 | rs2242593 | 0.118 | 1.000 | 0.290 | 6.44E-03 | 1.39E-01 |
| Substantia nigra | DRD2 | rs2242591 | 0.118 | 1.000 | 0.290 | 6.44E-03 | 1.39E-01 |
| Substantia nigra | DRD2 | rs6278 | 0.118 | 1.000 | 0.290 | 6.44E-03 | 1.39E-01 |
| Substantia nigra | DRD2 | rs1124491 | 0.118 | 1.000 | 0.290 | 6.44E-03 | 1.39E-01 |
| Substantia nigra | DRD2 | rs1079595 | 0.118 | 1.000 | 0.290 | 6.44E-03 | 1.39E-01 |
| Substantia nigra | DRD2 | rs1079594 | 0.118 | 1.000 | 0.290 | 6.44E-03 | 1.39E-01 |
| Substantia nigra | DRD2 | rs1076560 | 0.118 | 1.000 | 0.290 | 6.44E-03 | 1.39E-01 |
| Substantia nigra | DRD2 | rs2734848 | 0.754 | 1.000 | -0.286 | 7.33E-03 | 1.55E-01 |
| Substantia nigra | DRD2 | rs55900980 | 0.114 | 1.000 | 0.278 | 9.26E-03 | 1.83E-01 |
| Substantia nigra | DRD2 | rs2283265 | 0.114 | 1.000 | 0.278 | 9.26E-03 | 1.83E-01 |
| Substantia nigra | DRD2 | rs2075654 | 0.114 | 1.000 | 0.278 | 9.26E-03 | 1.83E-01 |
| Substantia nigra | DRD2 | rs1079727 | 0.114 | 1.000 | 0.278 | 9.26E-03 | 1.83E-01 |
| Substantia nigra | DRD2 | rs2734836 | 0.114 | 1.000 | 0.278 | 9.26E-03 | 1.83E-01 |
| Substantia nigra | DRD2 | rs1962262 | 0.114 | 1.000 | 0.278 | 9.26E-03 | 1.83E-01 |
| Substantia nigra | DRD2 | rs1079598 | 0.114 | 1.000 | 0.278 | 9.26E-03 | 1.83E-01 |
| Substantia nigra | DRD2 | rs1125393 | 0.118 | 1.000 | 0.278 | 9.26E-03 | 1.83E-01 |
| Substantia nigra | DRD2 | rs2471855 | 0.118 | 1.000 | 0.278 | 9.26E-03 | 1.83E-01 |
| Substantia nigra | DRD2 | rs2471851 | 0.114 | 1.000 | 0.276 | 9.67E-03 | 1.87E-01 |
| Substantia nigra | DRD2 | rs7350522 | 0.110 | 1.000 | 0.276 | 9.67E-03 | 1.87E-01 |
| Substantia nigra | DRD3 | rs78622784 | 0.044 | 1.000 | 0.617 | 4.92E-03 | 1.19E-01 |
| Substantia nigra | DRD3 | rs115852660 | 0.039 | 1.000 | 0.617 | 4.92E-03 | 1.19E-01 |
| Substantia nigra | SLC6A3 | rs3863145 | 0.246 | 1.000 | 0.467 | 9.53E-07 | 1.01E-03 |
| Substantia nigra | SLC6A3 | rs1809939 | 0.246 | 1.000 | 0.467 | 9.53E-07 | 1.01E-03 |
| Substantia nigra | SLC6A3 | rs3797200 | 0.246 | 1.000 | 0.467 | 9.53E-07 | 1.01E-03 |
| Substantia nigra | SLC6A3 | rs11564774 | 0.268 | 1.000 | 0.440 | 4.57E-06 | 2.00E-03 |
| Substantia nigra | SLC6A3 | rs1042098 | 0.285 | 1.000 | 0.440 | 4.65E-06 | 2.00E-03 |
| Substantia nigra | SLC6A3 | rs40184 | 0.399 | 1.000 | 0.347 | 4.12E-04 | 3.22E-02 |
| Substantia nigra | SLC6A3 | rs11133767 | 0.346 | 1.000 | 0.345 | 4.48E-04 | 3.45E-02 |
| Substantia nigra | SLC6A3 | rs72715526 | 0.204 | 0.991 | 0.292 | 3.38E-03 | 9.25E-02 |
| Substantia nigra | SLC6A3 | rs11133770 | 0.224 | 1.000 | 0.263 | 8.16E-03 | 1.68E-01 |
