# Supplementary material for: Expression of Dopamine-Related Genes in Four Human Brain Regions
Source: Brain Sci. 2020 Aug 18;10(8):567. doi: 10.3390/brainsci10080567 (PMC7465182; doi:10.3390/brainsci10080567)
Supplement: Supplementary file 1 [file brainsci-10-00567-s001.zip › Supplementary Table 2.docx]

| **Supplementary Table 2.** Demographic and phenotypic characteristics for participants (N=249). | | |
| --- | --- | --- |
| **Characteristic** | | **N(%)**  **or median (range)** |
| Sex | Female | 70 (28.11%) |
|  | Male | 179 (71.89%) |
| Race | Asian | 1 (0.4%) |
|  | Black | 22 (8.87%) |
|  | White | 225 (90.73%) |
| Age | | 61(55-66) |
| BMI | | 27.44(24.99-31.32) |
