# Supplementary material for: Expression of Dopamine-Related Genes in Four Human Brain Regions
Source: Brain Sci. 2020 Aug 18;10(8):567. doi: 10.3390/brainsci10080567 (PMC7465182; doi:10.3390/brainsci10080567)
Supplement: Supplementary file 1 [file brainsci-10-00567-s001.zip › Supplementary Table 3.docx]

| **Supplementary Table 3.** Association of gene expression with demographic and phenotypic characteristics, by tissue. | | | | | | | |
| --- | --- | --- | --- | --- | --- | --- | --- |
| **Tissue** | **Gene** | **N** | **Characteristic** | | **N(%)**  **or mean(SD)** | **Median_IQR or Spearman correlation coefficient** | ***p*-value** |
| Nucleus accumbens (basal ganglia) | *ANKK1* | 153 | Sex | Female | 45(29.4%) | 25(10~54) | 0.5373 |
|  |  |  |  | Male | 108(70.6%) | 22(10~47.25) |  |
|  |  |  | Race | Asian | - | - |  |
|  |  |  |  | Black | 16(10.5%) | 20(9.25~41.75) | 0.4748 |
|  |  |  |  | White | 136(89.5%) | 24(10~51) |  |
|  |  |  | Age | | 58.5(10.12) | 0.0124 | 0.8795 |
|  |  |  | BMI | | 27.47(4.01) | 0.0391 | 0.6318 |
|  | *DBH* | 170 | Sex | Female | 51(30%) | 20(12~37.5) | 0.16 |
|  |  |  |  | Male | 119(70%) | 17(8~31) |  |
|  |  |  | Race | Asian | 1(0.6%) | 11(11~11) | 0.0704 |
|  |  |  |  | Black | 16(9.5%) | 30.5(23.25~39) |  |
|  |  |  |  | White | 152(89.9%) | 17(9~31.25) |  |
|  |  |  | Age | | 58.29(10.08) | 0.0369 | 0.6331 |
|  |  |  | BMI | | 27.34(3.99) | -0.1358 | 0.0775 |
|  | *DRD1* | 145 | Sex | Female | 33(22.8%) | 134(67~249) | 0.6764 |
|  |  |  |  | Male | 112(77.2%) | 146(72.5~277.25) |  |
|  |  |  | Race | Asian | 1(0.7%) | 67(67~67) | 0.6656 |
|  |  |  |  | Black | 15(10.3%) | 114(92~254.5) |  |
|  |  |  |  | White | 129(89%) | 146(71~276) |  |
|  |  |  | Age |  | 58.71(10.17) | -0.1977 | 0.0171 |
|  |  |  | BMI |  | 27.28(3.94) | 0.046 | 0.5826 |
|  | *DRD2* | 177 | Sex | Female | 48(27.1%) | 271.5(99.75~553.5) | 0.1814 |
|  |  |  |  | Male | 129(72.9%) | 243(9~724) |  |
|  |  |  | Race | Asian | 1(0.6%) | 38(38~38) | 0.7017 |
|  |  |  |  | Black | 16(9.1%) | 374.5(30~755) |  |
|  |  |  |  | White | 159(90.3%) | 244(15~711.5) |  |
|  |  |  | Age |  | 58.18 (±10.11) | -0.0131 | 0.8631 |
|  |  |  | BMI |  | 27.48 (±4.13) | 0.0943 | 0.2117 |
|  | *DRD3* | 136 | Sex | Female | 35(25.7%) | 8(2.5~25.5) | 0.1847 |
|  |  |  |  | Male | 101(74.3%) | 13(3~34) |  |
|  |  |  | Race | Asian | - | - |  |
|  |  |  |  | Black | 13(9.6%) | 36(24~46) | 2e-04 |
|  |  |  |  | White | 123(90.4%) | 10(3~24) |  |
|  |  |  | Age |  | 58.35(10.53) | -0.1879 | 0.0285 |
|  |  |  | BMI |  | 27.61(3.78) | 0.0403 | 0.6417 |
|  | *DRD5* | 104 | Sex | Female | 29(27.9%) | 6(3~11) | 0.6679 |
|  |  |  |  | Male | 75(72.1%) | 6(3~10) |  |
|  |  |  | Race | Asian | 1(1%) | 2(2~2) | 0.3993 |
|  |  |  |  | Black | 12(11.5%) | 4(2.75~9.5) |  |
|  |  |  |  | White | 91(87.5%) | 6(3~10.5) |  |
|  |  |  | Age |  | 57.67(10.08) | -0.094 | 0.3423 |
|  |  |  | BMI |  | 27.28(4.03) | -0.0838 | 0.3977 |
| Substantia nigra | *ANKK1* | 80 | Sex | Female | 26(32.5%) | 8.5(4.25~18.75) | 0.2185 |
|  |  |  |  | Male | 54(67.5%) | 7.5(4~13) |  |
|  |  |  | Race | Asian | - | - |  |
|  |  |  |  | Black | 10(12.5%) | 8(6.25~13) | 0.6149 |
|  |  |  |  | White | 70(87.5%) | 7.5(3.25~15.5) |  |
|  |  |  | Age |  | 57.24(11.4) | -0.0326 | 0.7743 |
|  |  |  | BMI |  | 26.67(4.25) | 0.0779 | 0.492 |
|  | *DBH* | 91 | Sex | Female | 28(30.8%) | 10.5(6~19) | 0.5554 |
|  |  |  |  | Male | 63(69.2%) | 10(5~21.5) |  |
|  |  |  | Race | Asian | 1(1.1%) | 17(17~17) | 0.4204 |
|  |  |  |  | Black | 9(9.9%) | 22(6~23) |  |
|  |  |  |  | White | 81(89%) | 10(5~19) |  |
|  |  |  | Age |  | 57.13(11.37) | -0.1126 | 0.2881 |
|  |  |  | BMI |  | 26.53(4.28) | 0.0135 | 0.8991 |
|  | *DRD1* | 54 | Sex | Female | 13(24.1%) | 3(2~3) | 0.4863 |
|  |  |  |  | Male | 41(75.9%) | 2(1~4) |  |
|  |  |  | Race | Asian | - | - |  |
|  |  |  |  | Black | 7(13%) | 2(1.5~5) | 0.8531 |
|  |  |  |  | White | 47(87%) | 2(1~4) |  |
|  |  |  | Age |  | 57.41(12.83) | -0.2275 | 0.098 |
|  |  |  | BMI |  | 25.97(4.34) | 0.1368 | 0.3238 |
|  | *DRD2* | 87 | Sex | Female | 31(35.6%) | 32(5~225.5) | 0.6511 |
|  |  |  |  | Male | 56(64.4%) | 23.5(7~203.25) |  |
|  |  |  | Race | Asian | 1(1.1%) | 1(1~1) | 0.0474 |
|  |  |  |  | Black | 7(8%) | 207(23.5~358.5) |  |
|  |  |  |  | White | 79(90.8%) | 24(6~202) |  |
|  |  |  | Age |  | 58.06 (±10.92) | 0.1411 | 0.1924 |
|  |  |  | BMI |  | 26.76 (±4.24) | 0.211 | 0.0498 |
|  | *DRD5* | 51 | Sex | Female | 14(27.5%) | 6.5(3~17.75) | 0.0181 |
|  |  |  |  | Male | 37(72.5%) | 2(1~6) |  |
|  |  |  | Race | Asian | 1(2%) | 20(20~20) | 0.2208 |
|  |  |  |  | Black | 6(11.8%) | 1.5(1~5) |  |
|  |  |  |  | White | 44(86.3%) | 3(1.75~7.25) |  |
|  |  |  | Age |  | 55.76(11.9) | -0.1048 | 0.4644 |
|  |  |  | BMI |  | 26.51(4.37) | -0.1121 | 0.4334 |
|  | *SLC6A3* | 100 | Sex | Female | 28(28%) | 613(97.25~3402.25) | 0.0439 |
|  |  |  |  | Male | 72(72%) | 215.5(33.75~789) |  |
|  |  |  | Race | Asian | 1(1%) | 65(65~65) | 0.2405 |
|  |  |  |  | Black | 11(11%) | 1548(206.5~3567.5) |  |
|  |  |  |  | White | 88(88%) | 234.5(36.25~884.75) |  |
|  |  |  | Age |  | 57.78(11.23) | 0.0155 | 0.8784 |
|  |  |  | BMI |  | 26.59(4.4) | 0.158 | 0.1164 |
| Hippocampus | *ANKK1* | 118 | Sex | Female | 40(33.9%) | 5.5(3~11.5) | 0.9228 |
|  |  |  |  | Male | 78(66.1%) | 6(3~13.75) |  |
|  |  |  | Race | Asian | - | - |  |
|  |  |  |  | Black | 11(9.3%) | 4(4~6) | 0.3204 |
|  |  |  |  | White | 107(90.7%) | 6(3~14) |  |
|  |  |  | Age |  | 58.11(10.85) | 0.1614 | 0.0807 |
|  |  |  | BMI |  | 27.54(4.01) | 0.0595 | 0.5224 |
|  | *DBH* | 132 | Sex | Female | 42(31.8%) | 11.5(7~22.75) | 0.6179 |
|  |  |  |  | Male | 90(68.2%) | 12(5~22.5) |  |
|  |  |  | Race | Asian | - | - |  |
|  |  |  |  | Black | 12(9.1%) | 16(6.5~28.25) | 0.2957 |
|  |  |  |  | White | 120(90.9%) | 11.5(6~22) |  |
|  |  |  | Age |  | 58.92(10.29) | -0.0795 | 0.3646 |
|  |  |  | BMI |  | 27.58(3.97) | -0.1033 | 0.2385 |
|  | *DRD1* | 102 | Sex | Female | 27(26.5%) | 5(1.5~13.5) | 0.781 |
|  |  |  |  | Male | 75(73.5%) | 6(2.5~14) |  |
|  |  |  | Race | Asian | - | - |  |
|  |  |  |  | Black | 11(10.8%) | 7(1.5~14) | 0.7782 |
|  |  |  |  | White | 91(89.2%) | 6(2~13.5) |  |
|  |  |  | Age |  | 59.18(10.79) | -0.0939 | 0.3479 |
|  |  |  | BMI |  | 27.37(3.97) | -0.0737 | 0.4617 |
|  | *DRD2* | 119 | Sex | Female | 39(32.8%) | 10(4~18.5) | 0.2688 |
|  |  |  |  | Male | 80(67.2%) | 9(3~14) |  |
|  |  |  | Race | Asian | - | - |  |
|  |  |  |  | Black | 9(7.6%) | 13(10~17) | 0.1323 |
|  |  |  |  | White | 110(92.4%) | 8.5(3~16) |  |
|  |  |  | Age |  | 58.32 (±10.51) | 0.0263 | 0.7766 |
|  |  |  | BMI |  | 27.77 (±4.11) | 0.0997 | 0.2805 |
|  | *DRD5* | 89 | Sex | Female | 27(30.3%) | 6(2~10) | 0.0502 |
|  |  |  |  | Male | 62(69.7%) | 10(5~27.75) |  |
|  |  |  | Race | Asian | - | - |  |
|  |  |  |  | Black | 12(13.5%) | 7(4.5~17.5) | 0.7226 |
|  |  |  |  | White | 77(86.5%) | 9(3~23) |  |
|  |  |  | Age |  | 57.72(9.93) | -0.2648 | 0.0122 |
|  |  |  | BMI |  | 27.45(4.09) | 0.1291 | 0.228 |
| Prefrontal cortex | *ANKK1* | 127 | Sex | Female | 37(29.1%) | 8(4~10) | 0.338 |
|  |  |  |  | Male | 90(70.9%) | 6.5(3~10) |  |
|  |  |  | Race | Asian | - | - |  |
|  |  |  |  | Black | 14(11%) | 5.5(2.5~7.75) | 0.4871 |
|  |  |  |  | White | 113(89%) | 7(3~10) |  |
|  |  |  | Age |  | 58.75(10.19) | -0.0114 | 0.8991 |
|  |  |  | BMI |  | 27.65(4.08) | 0.0745 | 0.4051 |
|  | *DBH* | 161 | Sex | Female | 43(26.7%) | 39(20~63.5) | 0.1199 |
|  |  |  |  | Male | 118(73.3%) | 32.5(13.25~50.75) |  |
|  |  |  | Race | Asian | - | - |  |
|  |  |  |  | Black | 16(9.9%) | 56.5(30.5~93) | 0.0184 |
|  |  |  |  | White | 145(90.1%) | 32(17~51) |  |
|  |  |  | Age |  | 58.73(10) | 0.0011 | 0.9894 |
|  |  |  | BMI |  | 27.59(4.03) | -0.0614 | 0.4389 |
|  | *DRD1* | 123 | Sex | Female | 28(22.8%) | 22(12.75~49) | 0.8825 |
|  |  |  |  | Male | 95(77.2%) | 24(15~41) |  |
|  |  |  | Race | Asian | - | - |  |
|  |  |  |  | Black | 15(12.2%) | 25(17~39.5) | 0.6877 |
|  |  |  |  | White | 108(87.8%) | 23(13~44.5) |  |
|  |  |  | Age |  | 59.5(10.07) | -0.2212 | 0.014 |
|  |  |  | BMI |  | 27.34(4.04) | -0.0309 | 0.734 |
|  | *DRD2* | 124 | Sex | Female | 38(30.6%) | 9(5.25~13.75) | 0.5024 |
|  |  |  |  | Male | 86(69.4%) | 8(3~15) |  |
|  |  |  | Race | Asian | - | - |  |
|  |  |  |  | Black | 11(8.9%) | 8(5~16) | 0.6471 |
|  |  |  |  | White | 113(91.1%) | 9(4~14) |  |
|  |  |  | Age |  | 59.33 (±9.63) | -0.0903 | 0.3186 |
|  |  |  | BMI |  | 27.51 (±4.15) | 0.0804 | 0.3747 |
|  | *DRD5* | 103 | Sex | Female | 28(27.2%) | 15.5(7.25~27.75) | 0.3312 |
|  |  |  |  | Male | 75(72.8%) | 18(7~33.5) |  |
|  |  |  | Race | Asian | - | - |  |
|  |  |  |  | Black | 11(10.7%) | 19(12~29.5) | 0.6307 |
|  |  |  |  | White | 92(89.3%) | 16(7~32) |  |
|  |  |  | Age |  | 58.67(9.16) | -0.3179 | 0.0011 |
|  |  |  | BMI |  | 27.35(4.23) | -0.0106 | 0.9155 |
