# Supplementary figures and images for: Expression of Dopamine-Related Genes in Four Human Brain Regions
Source: Brain Sci. 2020 Aug 18;10(8):567. doi: 10.3390/brainsci10080567 (PMC7465182; doi:10.3390/brainsci10080567)

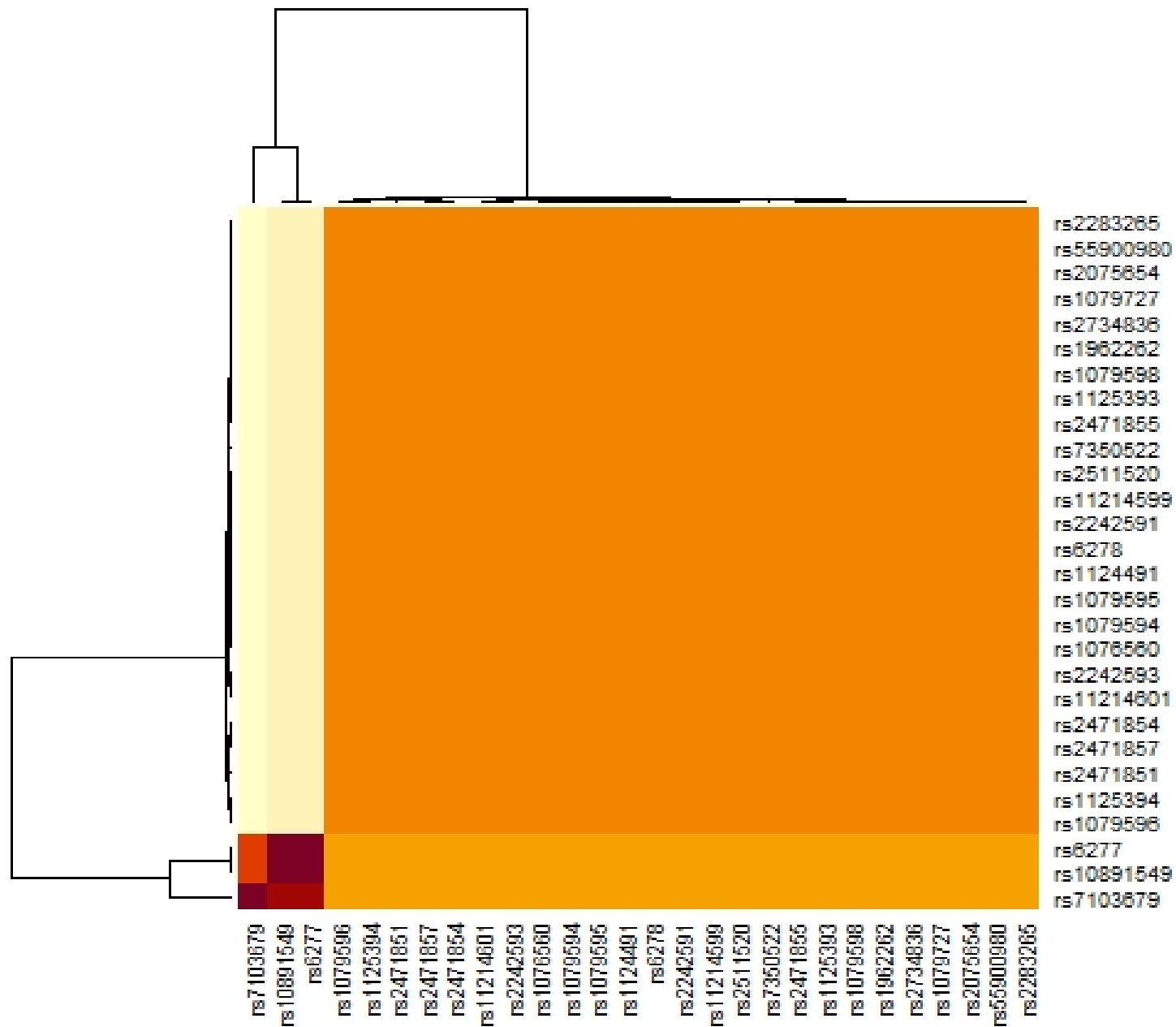

**Supplementary Figure 1.** Linkage disequilibrium structure for 3 haplotype blocks of *DRD2*.

Supplement: Supplementary file 1 [file brainsci-10-00567-s001.zip › Supplementary Figure 1.pdf]
